# Hydronopylformamides: Modification of the Naturally Occurring Compound (-)-β-Pinene to Produce Insect Repellent Candidates against *Blattella germanica*

**DOI:** 10.3390/molecules22061004

**Published:** 2017-06-16

**Authors:** Shengliang Liao, Yan Liu, Hongyan Si, Zhuanquan Xiao, Guorong Fan, Shangxing Chen, Peng Wang, Zongde Wang

**Affiliations:** 1 College of Forestry, Jiangxi Agricultural University, Camphor Tree Engineering and Technology Research Center of State Forestry Administration and Jiangxi Province, Nanchang 330045, Jiangxi, China; liaosl@jxau.edu.cn (S.L.); sihy@jxau.edu.cn (H.S.); fgr008@126.com (G.F.); csxing@126.com (S.C.); pengwang1981@126.com (P.W.); 2 Yichun Hydrological Bureau of Jiangxi Province, Yichun 336000, Jiangxi, China; lyjy89@163.com; 3 College of Chemistry and Chemical Engineering, Jiangxi Normal University, Nanchang 330027, China; kingleft13@163.com

**Keywords:** β-pinene, synthesis, *Blattella germanica*, repellent

## Abstract

The development of a novel repellent plays an important role in the integrated control of *Blattella germanica*. A series of novel hydronopylformamides derivatives were synthesized from a naturally occurring compound (-)-β-pinene. The structures of these hydronopylformamides derivatives were characterized by Fourier transform infrared spectroscopy (FTIR), nuclear magnetic resonance spectroscopy (^1^H-NMR and ^13^C-NMR), and electron impact mass spectrometry (EI-MS). Repellency of these hydronopylformamides derivatives against *Blattella germanica* was evaluated by the using petri dish arena method. The results showed that four derivatives (compounds **8a**, **8b**, **8c** and **8e**) exhibited repellency against *Blattella germanica* at a concentration of 20 mg/mL. Compound **8a** was the most active compound among these derivatives, where the repelling ratios of compound **8a** against *Blattella germanica* were 66.10%, 50.46%, 48.26%, at concentrations of 20 mg/mL, 10 mg/mL, and 5 mg/mL, respectively. In addition, compound **8a** showed better repellency than the traditional insect repellent *N*, *N*-diethyl-3-methylbenzamide (DEET), which indicated that compound **8a** had a good application prospect in the prevention of *Blattella germanica*. This research hopes to promote the value-added utilization of (-)-β-pinene and the development of novel German cockroach repellents.

## 1. Introduction

*Blattella germanica* is a common household insect pest worldwide, which may disseminate many serious human diseases [1,2]. Therefore, the control of *Blattella germanica* is of great importance. Currently, the control of *Blattella germanica* populations depend on continued application of insecticides. Although the application of insecticides is efficient, the increasing resistance of *Blattella germanica* to insecticides seriously threatens the ongoing management of *Blattella germanica* [3]. Furthermore, the negative effects of insecticides in the indoor environment have also caused much concern. Hence, these issues have highlighted the need for the development of an integrated strategy for the control of *Blattella germanica*. Insect repellents, which would discourage *Blattella germanica* and let them fly or crawl away, were regarded as a good option for the integrated control of *Blattella germanica*.

Naturally occurring compounds are ideal raw materials for the development of insect repellents as they are safer and pose less risk to the environment [4]. Many reports have described the repellency of naturally occurring compounds against cockroaches [5,6,7,8]; however, the repellency and duration times of naturally occurring compounds were usually unsatisfactory. To improve the repellency and duration times, the chemical modification of naturally occurring compounds was a feasible method. The preparation of novel repellents by modifying naturally occurring compounds has attracted much attention in recent years.

(-)-β-pinene is a main component of turpentine, which is an essential oil obtained from live trees, mainly pines. Previous research has found that (-)-β-pinene exhibited a mild repellency against *Blattella germanica*, and that some synthetic derivatives of β-pinene exerted better repellency [9]. These findings indicated that novel repellent candidates might be screened out from the derivatives of (-)-β-pinene. In order to screen novel repellent candidates against *Blattella germanica*, a series of novel (-)-β-pinene derivatives were designed and synthesized, and the repellency of these derivatives against *Blattella germanica* was evaluated in this research.

## 2. Results and Discussion

### 2.1. Chemistry

**Figure 1:**
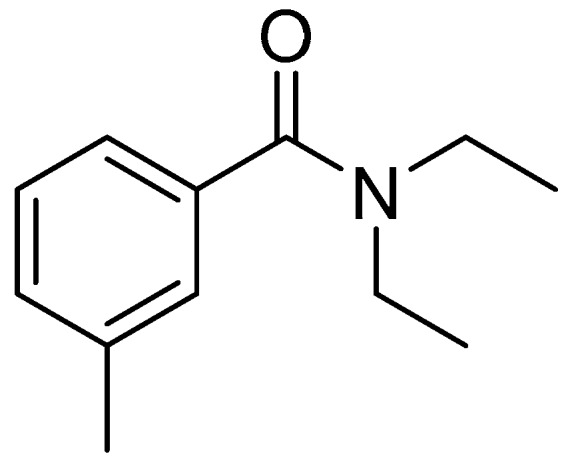
Molecular structure of *N*, *N*-diethyl-m-toluamide (DEET).

Since *N*,*N*-diethyl-m-toluamide (DEET, Figure 1) was discovered and used as insect repellent, the repellency of amides was the subject of constant attention. DEET was synthesized in 1953, and by far it was and still is, the most widely used insect repellent [10]. However, some toxic effects of DEET have since been disclosed, such as underlying neurological disorders, neurotoxicity, allergic reactions, etc. [11,12,13,14]. Hence, the development of safer amide repellents is important and still in high demand.

**Figure 2:**
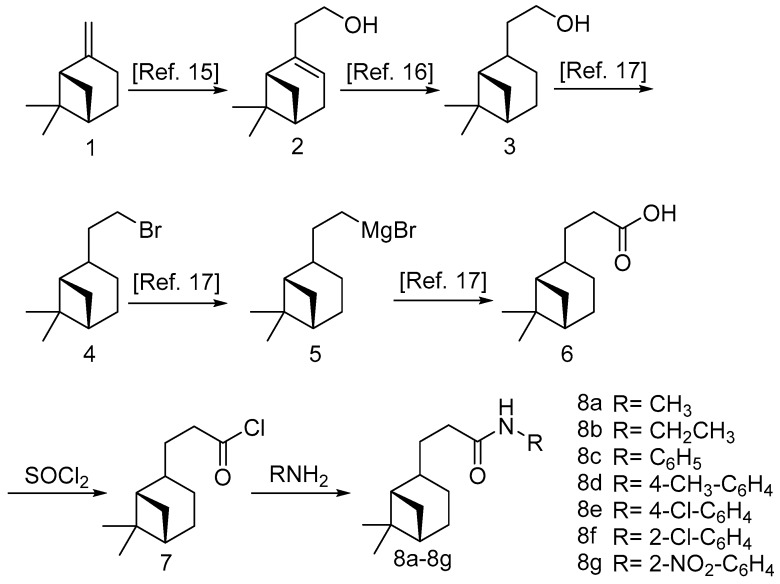
Synthesis routine of hydronopylformamides **8a**–**8g**.

In this work, a series of amides derivatives of (-)-β-pinene were synthesized (Figure 2). (-)-β-pinene was first transferred into nopol via a Prins reaction [15], and then hydronopol was obtained by the hydrogenation of nopol [16]. After a Grignard reaction, hydronopol was converted to hydronopyl formic acid [17]. Hydronopylformamides were prepared by the amidation reactions of a series of amines and the acyl chloride of hydronopyl formic acid. Generally, amides are typically synthesized from the union of carboxylic acids and amines; however, it is usually necessary to activate the carboxylic acids in advance as the carboxylic acids and amines do not spontaneously combine at mild conditions. There are many reagents that can be used to activate the carboxylic acids, such as carbodiimides, salts of 1*H*-benzotriazole, acid halides, etc. [18]; nevertheless, the most common acid halide, thionyl chloride, was selected as the activator in this work due to its high-efficiency, low-cost and post-treated convenience.

The structures of these hydronopylformamides were characterized by Fourier transform infrared spectroscopy (FTIR), nuclear magnetic resonance spectroscopy (^1^H-NMR and ^13^C-NMR), and electron impact mass spectrometry (EI-MS) Taking compound **8e** as an example, in the IR spectrum, the absorption band at 3276 cm^−1^ and 3240 cm^−1^ was assigned to the -NH group; the absorption bands at 2983–2858 cm^−1^ were attributed to -CH_2_-, and -CH-; the absorption bands at 1655 cm^−1^ and 1595–1491 cm^−1^ represented the existence of -C=O and aromatic ring; the absorption bands at 1540 was assigned to C-N; and the absorption band at 834 cm^−1^ was attributed to 1,4-disubstituted benzene. In the ^1^H-NMR spectrum of compound **8e**, the signal appearing as a singlet at about 7.68 ppm was assigned to the proton of the acyl amino group. The signals attributed to four aromatic protons of benzene rings were observed at 7.47 ppm and 7.25 ppm. Other signals were assigned to the protons in the pinane skeleton. In the ^13^C-NMR spectrum of compound **8e**, the characteristic C=O peaks related to the acyl amino group were observed at 171.92 ppm; the signals of aromatic carbons appeared at 136.55–121.07 ppm; and the characteristic C-Cl peaks related to the benzene ring were observed at 129.02 ppm. The signals of aliphatic groups appeared between 46.12 ppm and 22.13 ppm. In the EI-MS spectrum of compound **8e**, the signals at 305 [M^+^] and 307 [M^+^ + 2] indicated that the molecular weight of **8e** was 305. These results showed that the characterization data were in full agreement with the proposed structures.

### 2.2. Biological Activity

The repellency of hydronopylformamides at the concentration of 20 mg/mL against *Blattella germanica* are listed in Table 1. From this, it can be seen that compounds **8a**, **8b**, **8c**, and **8e** exhibited repellency against *Blattella germanica*, and compound **8a** with a repelling rate of 67.06% against *Blattella germanica* was the most active compound. Compounds **8d**, **8f**, and **8g** did not show any repellency against *Blattella germanica*; furthermore, compound **8g** exhibited slight attractive activity against *Blattella germanica*. 

The influence of substituent groups and melting point of these hydronopylformamides on their biological activity was also discussed. As shown in Table 1, compound **8a** (with a methyl group in the N atom) exhibited the best repellency; however, other derivatives with bigger substituent groups in the N atom presented weaker repellency, and these results revealed that for the hydronopylformamides, a smaller substituent group in the N atom was more beneficial for repellency. When the melting points of hydronopylformamides were lower than 100 °C, the hydronopylformamides exhibited biological activity either as repellency (compounds **8a**, **8b** and **8c**) or attractive activity (compound **8g**). However, when the melting points of hydronopylformamides were higher than 100 °C, the hydronopylformamides showed weak biological activity. 

Furthermore, the repellency of compound **8a** and DEET against *Blattella germanica* at three different concentrations were tested, and the results are summarized in Table 2. Table 2 shows that the repellency of compound **8a** was better than that of DEET at all tested concentrations, including 20 mg/mL, 10 mg/mL, and 5 mg/mL. It is generally known that DEET is a broad-spectrum repellent that serves an effective defensive role against many insects, such as cockroaches, mosquitos, flies, etc. [10]. In this research, compound **8a** exhibited better repellency than DEET against the cockroach *Blattella germanica*, which indicated that compound **8a** was a good application prospect in the prevention of *Blattella germanica*. Further research is required to evaluate the repellency of compound **8a** against more cockroach species.

## 3. Materials and Methods

### 3.1. General

(-)-β-pinene was purchased from the spice company Jiangxi Jishui Hongda Natural Perfume Co., Ltd., Ji’an, China, and other reagents were of reagent grade. Melting points were determined using X-4B melting point apparatus (Shanghai Precision & Scientific Instrument Co., Ltd., Shanghai, China) and were uncorrected. IR spectra were recorded on a Nicolet IR6700 FT-IR spectrometer (Nicolet, Waltham, MA, USA). ^1^H-NMR and ^13^C-NMR spectra were recorded on a Bruker AVANCE 400 NMR spectrometer (Bruker, Karlsruhe, Germany) at 400 MHz and 100 MHz, respectively, using CDCl_3_ as the solvent and TMS as the internal standard. EI-MS were recorded on a Clarus 600C GC/MS spectrometer (PerkinElmer, Waltham, MA, USA). The purity of compounds was detected by Fuli GC9790 gas chromatography (Zhejiang Fuli analysis instrument Co., Ltd., Wenling, China). *Blattella germanica* were obtained from Jiangxi Hilltop Chemical Industrial Co., Ltd., Zhangshu, China.

### 3.2. Synthesis

#### 3.2.1. Synthesis of Nopol (**2**), Hydronopol (**3**), Hydronopyl Bromide (**4**), Hydronopyl Formic Acid (**6**)

Based on previous studies described in References [15,16,17], nopol (**2**), hydronopol (**3**), hydronopyl bromide (**4**), and hydronopyl formic acid (**6**) were prepared.

#### 3.2.2. Synthesis of Hydronopylformamides (**8a**–**8g**) (Taking Compound **8e** as An Example)

Thionyl chloride (1.785 g, 15 mmol) was slowly added to a solution of hydronopyl formic acid (1.962 g, 10 mmol) in anhydrous methylene chloride (10 mL), and stirred at 50 °C for 3 h. Next, the mixture was concentrated under reduced pressure to remove the solvent and excess thionyl chloride. The residue was dissolved in dried methylene chloride (10 mL), and added dropwise into a solution of 4-chloroaniline (1.914 g, 15 mmol) and triethylamine (1.518 g, 15 mmol) in anhydrous methylene chloride (10 mL), and the mixture was stirred at 50 °C until the reaction was complete (as per the thin-layer chromatography and gas chromatography analysis). The mixture was cooled, diluted with 80 mL methylene chloride, and treated with 20 mL 5% aqueous solution of HCl, washed with a saturated aqueous solution of sodium chloride and dried over anhydrous sodium sulfate, after which the solvent was removed under reduced pressure. Further purification was accomplished by silica gel chromatography to give pure products **8e**.

The structures of these hydronopylformamides derivatives were characterized by Fourier transform infrared spectroscopy (FTIR), nuclear magnetic resonance spectroscopy (^1^H-NMR and ^13^C-NMR), and electron impact mass spectrometry (EI-MS), and the spectrums were included in the Supplementary Materials available online.

*3-((1S,5S)-6,6-Dimethylbicyclo[3.1.1]heptan-2-yl)-N-methylpropanamide* (compound **8a**). White solid, m.p. 38.0–38.5 °C, yield 70.4%, GC purity 97.5%; IR ν (cm^−1^): 3294, 2982, 2936, 2986, 2861, 1645, 1561, 1468, 1383, 1366; ^1^H-NMR (400 MHz, CDCl_3_) δ (ppm): 5.65 (1H, br), 2.78 (3H, s), 2.29 (1H, m), 2.17–2.10 (2H, m), 1.91–1.82 (6H, m), 1.69 (2H, m), 1.43 (1H, m), 1.15 (3H, s), 0.98 (3H, s), 0.83 (1H, d, *J* = 8 Hz); ^13^C-NMR (100 MHz, CDCl_3_) δ (ppm): 173.969, 46.100, 41.356, 41.127, 38.629, 35.238, 33.630, 33.371, 28.110, 26.330, 26.243, 23.226, 22.139; MS *m*/*z* (%RA): 53(14), 55(32), 58(57), 67(30), 69(24), 73(100), 74(21), 77(13), 79(22), 81(24), 82(20), 83(13), 86(71), 87(10), 91(12), 93(15), 95(13), 107(8), 109(7), 112(6), 121(11), 123(6), 127(15), 128(16), 135(6), 136(17), 137(28), 138(4), 140(13), 153(6), 154(4), 166(15), 178(7), 180(2), 194(4), 209(4), 210(0.6).

*3-((1S,5S)-6,6-Dimethylbicyclo[3.1.1]heptan-2-yl)-N-ethylpropanamide* (compound **8b**). Light yellow solid, m.p. 41.5–42.2 °C, yield 61.0%, GC purity 99.1%; IR ν (cm^−1^): 3285, 2976, 2934, 2901, 2864, 1643, 1553, 1468, 1383, 1366; ^1^H-NMR (400 MHz, CDCl_3_) δ (ppm): 5.72 (1H, s), 2.28 (1H, m), 2.16~2.05 (2H, m), 1.92–1.79 (6H, m), 1.69 (2H, m), 1.43 (1H, m), 1.14 (3H, s), 1.10 (3H, t), 0.97 (3H, s), 0.82 (1H, d, *J* = 8 Hz); ^13^C-NMR (100 MHz, CDCl_3_) δ (ppm): 173.139, 46.089, 41.346, 41.144, 38.594, 35.358, 34.198, 33.612, 33.336, 28.075, 26.302, 23.183, 22.115, 14.815; MS *m*/*z* (%RA): 53(10), 55(28), 67(26), 69(19), 72(60), 77(11), 79(17), 81(19), 82(13), 83(11), 87(100), 88(15), 91(10), 93(11), 95(10), 100(56), 101(7), 107(6), 109(6), 121(7), 123(5), 126(4), 136(7), 137(19), 141(12), 142(9), 154(12), 167(3), 168(3), 178(4), 180(12), 208(3), 223(5), 224(0.8).

*3-((1S,5S)-6,6-Dimethylbicyclo[3.1.1]heptan-2-yl)-N-phenylpropanamide* (compound **8c**). White solid, m.p. 84.7–85.7 °C, yield 67.9%, GC purity 97.7%; IR ν (cm^−1^): 3285, 3253, 2986, 2972, 2915, 2878, 2857, 1658, 1598, 1499, 1550, 1466, 1358, 754, 691; ^1^H-NMR (400 MHz, CDCl_3_) δ (ppm): 7.56 (1H, br), 7.54 (2H, d), 7.29 (2H, t), 7.09 (1H, t), 2.35–2.30 (3H, m), 1.97–1.79 (8H, m), 1.49–1.45 (1H, m), 1.17 (3H, s), 1.00 (3H, s), 0.86 (1H, d, *J* = 12 Hz); ^13^C-NMR (100 MHz, CDCl_3_) δ (ppm): 171.771, 138.011, 128.870, 124.453, 119.785, 46.148, 41.363, 41.057, 38.642, 36.330, 33.633, 33.193, 28.101, 26.321, 23.224, 22.134; MS *m*/*z* (%RA): 53(6), 55(15), 67(12), 69(11), 77(15), 79(10), 81(8), 92(34), 93(100), 94(12), 95(6), 107(4), 109(2), 120(5), 134(20), 135(66), 136(8), 137(5), 148(24), 189(2), 202(3), 228(2), 271(6), 272(1.1).

*3-((1S,5S)-6,6-Dimethylbicyclo[3.1.1]heptan-2-yl)-N-(p-tolyl)propanamide* (compound **8d**). White solid, m.p. 125.1–126.3 °C, yield 58.9%, GC purity 98.5%; IR ν (cm^−1^): 3278, 3242, 2985, 2938, 2902, 2858, 1650, 1601, 1511, 1540, 1459, 1381, 1366, 816; ^1^H-NMR (400 MHz, CDCl_3_) δ (ppm): 7.61 (1H, s), 7.40 (2H, d, *J* = 8 Hz), 7.09 (2H, d, *J* = 8 Hz), 2.36–2.26 (3H, m), 2.30 (3H, s), 1.96–1.76 (8H, m), 1.48~1.42 (1H, m), 1.18 (3H, s), 1.00 (3H, s), 0.85 (1H, d, *J* = 8 Hz); ^13^C-NMR (100 MHz, CDCl_3_) δ (ppm): 171.726, 135.443, 133.580, 129.309, 119.900, 46.122, 41.324, 41.032, 38.612, 36.227, 33.625, 33.220, 28.875, 26.309, 23.204, 22.091; MS *m*/*z* (%RA): 53(5), 55(12), 67(9), 69(9), 77(10), 79(13), 81(6), 91(12), 93(8), 95(4), 105(16), 106(52), 107(100), 108(13), 120(3), 134(4), 135(3), 137(2), 148(11), 149(62), 150(5), 162(19), 163(2), 216(3), 242(2), 285(7), 286(1.2).

*N-(4-Chlorophenyl)-3-((1S,5S)-6,6-dimethylbicyclo[3.1.1]heptan-2-yl)propanamide* (compound **8e**). White solid, m.p. 123.2–124.2 °C, yield 60.2%, GC purity 99.9%; IR ν (cm^−1^): 3276, 3240, 2983, 2939, 2905, 2858, 1655, 1595, 1491, 1540, 1467, 1398, 1360, 834, 1092; ^1^H-NMR (400 MHz, CDCl_3_) δ (ppm): 7.68 (1H, s), 7.47 (2H, d, *J* = 8 Hz), 7.25 (2H, d, *J* = 8 Hz), 2.37–2.26 (3H, m), 1.95–1.77 (8H, m), 1.47–1.41 (1H, m), 1.17 (3H, s), 0.99 (3H, s), 0.85 (1H, d, *J* = 8 Hz); ^13^C-NMR (100 MHz, CDCl_3_) δ (ppm): 171.919, 136.549, 129.018, 128.874, 121.073, 46.124, 41.319, 41.051, 38.638, 33.636, 33.132, 28.092, 26.299, 23.229, 22.132; MS *m*/*z* (%RA): 53(7), 55(22), 67(17), 69(17), 77(8), 79(13), 81(13), 82(7), 91(15), 93(12), 95(9), 105(3), 107(6), 121(5), 123(3), 126(25), 127(100), 128(15), 129(29), 130(3), 137(7), 168(7), 169(49), 170(6), 171(16), 182(16), 184(6), 223(2), 225(0.6), 262(2), 264(1), 305(4.4), 306(0.7), 307(1.6), 308(0.3).

*N-(2-Chlorophenyl)-3-((1S,5S)-6,6-dimethylbicyclo[3.1.1]heptan-2-yl)propanamide* (compound **8f**). White solid, m.p. 108.8–109.4 °C, yield 64.2%, GC purity 99.2%; IR ν (cm^−1^): 3230, 2977, 2934, 2909, 2874, 1657, 1585, 1533, 1482, 1532, 1441, 1383, 1365, 1063, 756; ^1^H-NMR (400 MHz, CDCl_3_) δ (ppm): 8.38 (1H, d, *J* = 8 Hz), 7.64 (1H, s), 7.35 (1H, m), 7.26 (1H, m), 7.02 (1H, m), 2.44–2.31 (3H, m), 2.06–1.81 (8H, m), 1.53–1.47 (1H, m), 1.19 (3H, s), 1.03 (3H, s), 0.88 (1H, d, *J* = 8 Hz); ^13^C-NMR (100 MHz, CDCl_3_) δ (ppm): 171.484, 134.617, 128.868, 127.673, 124.578, 121.532, 46.087, 41.385, 40.911, 38.676, 36.532, 33.600, 33.060, 28.116, 26.319, 23.235, 22.156; MS *m*/*z* (%RA): 53(9), 55(27), 67(20), 69(21), 77(11), 79(17), 81(16), 82(9), 83(6), 91(12), 93(18), 95(11), 99(5), 105(3), 107(9), 119(3), 126(8), 127(100), 128(10), 129(35), 130(3), 134(68), 135(9), 136(8), 137(10), 148(3), 154(4), 169(46), 171(15), 182(22), 184(7), 224(3), 236(3), 250(1), 262(2), 264(2), 270(2), 305(5.1), 306(1), 307(1.7), 308(0.3).

*3-((1S,5S)-6,6-Dimethylbicyclo[3.1.1]heptan-2-yl)-N-(2-nitrophenyl)propanamide* (compound **8g**). Yellow solid, m.p. 54.3–55.3 °C, yield 57.0%, GC purity 98.8%; IR ν (cm^−1^): 3242, 2977, 2939, 2910, 2856, 1663, 1608, 1592, 1491, 1538, 1518, 1466, 1365, 1346, 779; ^1^H-NMR (400 MHz, CDCl_3_) δ (ppm): 10.37 (1H, s), 8.78 (1H, d, *J* = 8 Hz), 8.19 (1H, d, *J* = 8 Hz), 7.62 (1H, m), 7.15 (1H, m), 2.47–2.32 (3H, m), 2.00–1.84 (8H, m), 1.49 (1H, m), 1.17 (3H, s), 1.02 (3H, s), 0.87 (1H, d, *J* = 8 Hz); ^13^C-NMR (100 MHz, CDCl_3_) δ (ppm): 172.373, 136.038, 135.927, 134.970, 125.642, 122.937, 122.021, 45.967, 41.292, 40.809, 38.630, 37.215, 33.539, 32.831, 28.051, 26.051, 23.197, 22.080; MS *m*/*z* (%RA): 53(25), 54(14), 55(100), 65(23), 67(76), 68(11), 69(72), 77(31), 78(12), 79(67), 80(20), 81(65), 82(48), 83(13), 90(15), 91(46), 92(31), 93(69), 94(11), 95(43), 107(5), 119(35), 120(7), 121(34), 122(38), 123(33), 124(8), 131(15), 132(20), 133(43), 134(16), 135(29), 136(10), 137(8), 138(73), 139(15), 145(11), 146(9), 147(15), 148(19), 149(10), 150(12), 151(18), 159(5), 161(5), 163(7), 172(9), 173(10), 177(6), 186(11), 193(13), 199(5), 211(3), 225(5), 239(5), 281(19), 282(3), 298(3), 316(1.4), 317(0.2).

### 3.3. Biological Assay

Repellency of novel hydronopylformamides against *Blattella germanica* was evaluated by the methods reported in the literatures [19]. Seven hydronopylformamides and DEET were dissolved in ethanol or acetone to prepare solutions with an initial concentration of 20 mg/mL. A piece of round filter paper (15.0 cm) was cut in half, one half was treated with 1.5 mL of the tested compound solution, and the other half was treated with 1.5 mL of ethanol or acetone. The two halves of the filter paper were dried for 5 min before being placed into a 15 cm petri dish arena. The walls of the petri dish were treated in advance with a mixture of Vaseline and paroline to prevent the insect from escaping. One cockroach at a time was introduced and left to acclimatize for 5 min. After 5 minutes, the number of seconds the cockroach spent on the treated or untreated side out of a total of 300 s (5 min) was timed with two stopwatches. The filter paper halves and cockroaches were only used once. Each test was replicated 10 times. All tests were run under overhead florescent lighting at an ambient temperature (20–25 °C) and humidity (50–70% RH). Repellency was calculated as per Equation (1).

Repelling ratio = (T1 − T2)/300 × 100%(1)

where T1 was the number of seconds spent on the untreated side; and T2 was the number of seconds spent on the treated side.

## 4. Conclusions

The repellency of seven novel hydronopylformamides derivatives of (-)-β-pinene (compound **8a**–**8g**) was evaluated against *Blattella germanica*. The results showed that compounds **8a**, **8b**, **8c** and **8e** exhibited repellency against *Blattella germanica*, and compound **8a** was the most active. Furthermore, the repellency of compound **8a** was better than that of DEET at all tested concentrations, including 20 mg/mL, 10 mg/mL, and 5 mg/mL. This research hopes to contribute to the value-added utilization of β-pinene, and the development of novel German cockroach repellents.

## Figures and Tables

**Table 1:** Repellency of hydronopylformamides against *Blattella germanica* at a concentration of 20 mg/mL.

Compounds	R	Melting Point	Repelling Rate ± Standard Error (%)
**8a**	CH_3_	38.0–38.5	67.06 ± 3.07
**8b**	CH_2_CH_3_	41.5–42.2	38.89 ± 3.61
**8c**	C_6_H_5_	84.7–85.7	41.19 ± 6.05
**8d**	4-CH_3_-C_6_H_4_	125.1–126.3	−1.83 ± 5.18
**8e**	4-Cl-C_6_H_4_	123.2–124.2	8.45 ± 7.81
**8f**	2-Cl-C_6_H_4_	108.8–109.4	−5.48 ± 9.97
**8g**	2-NO_2_-C_6_H_4_	54.3–55.3	−23.59 ± 6.67
Ethanol	-	-	0.39 ± 1.18
Acetone	-	-	0.71 ± 1.42
DEET	-	−33.0	54.77 ± 6.61

**Table 2:** Repellency of compound **8a** and DEET against *Blattella germanica* at three different concentrations.

Compounds	Repelling Rate ± Standard Error (%)		
	20 mg/mL	10 mg/mL	5 mg/mL
**8a**	67.06 ± 3.07	50.46 ± 5.07	48.26 ± 5.18
DEET	54.77 ± 6.61	34.02 ± 4.41	29.56 ± 6.48

## Supplementary Materials

The following are available online: FTIR, NMR and GC-MS spectrums of **8a**–**8g**.
