# Supplementary material for: Hydronopylformamides: Modification of the Naturally Occurring Compound (-)-β-Pinene to Produce Insect Repellent Candidates against Blattella germanica
Source: Molecules. 2017 Jun 16;22(6):1004. doi: 10.3390/molecules22061004 (PMC6152770; doi:10.3390/molecules22061004)
Supplement: Supplementary file 1 [file molecules-22-01004-s001.pdf]

FTIR Spectrums of compound 8a-8g

Compound 8a

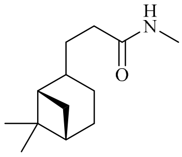

3-((1S,5S)-6,6-dimethylbicyclo[3.1.1]heptan-2-yl)-N-methylpropanamide

FTIR of compound 8a

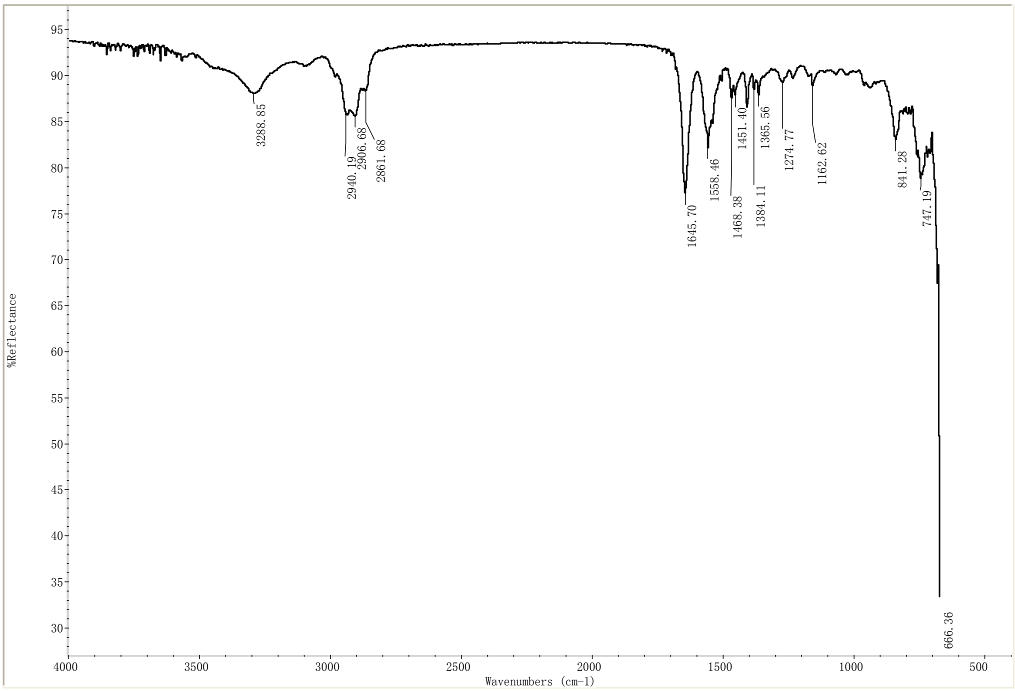

Compound 8b

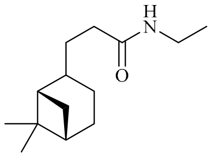

3-((1S,5S)-6,6-dimethylbicyclo[3.1.1]heptan-2-yl)-N-ethylpropanamide

FTIR of compound 8b

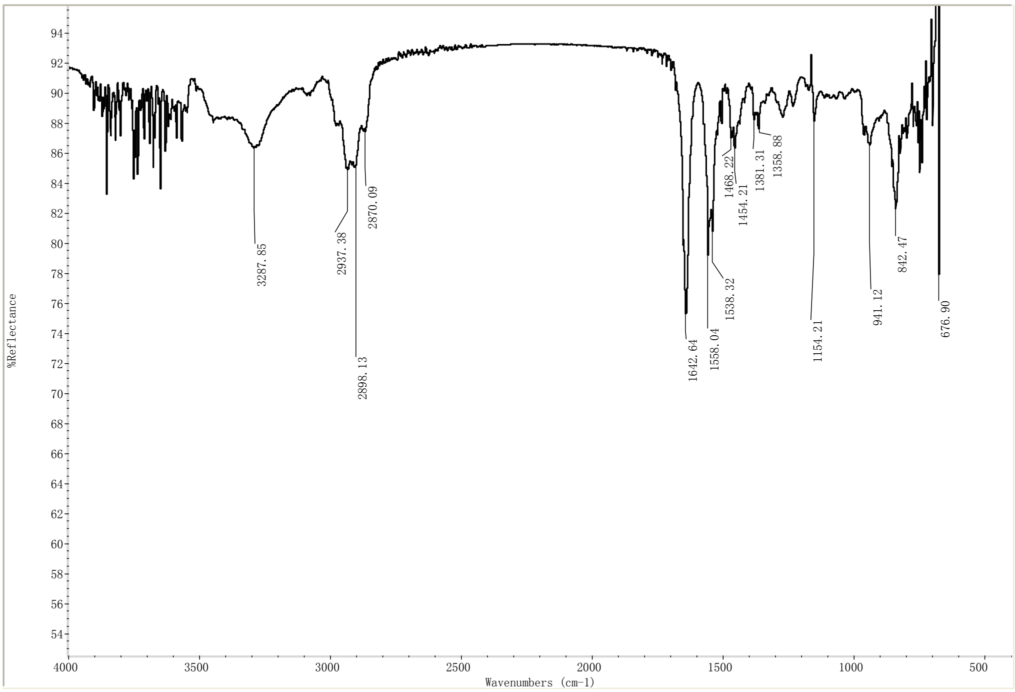

Compound 8c

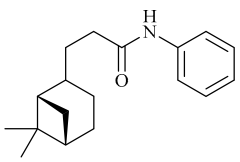

3-((1S,5S)-6,6-dimethylbicyclo[3.1.1]heptan-2-yl)-N-phenylpropanamide

FTIR of compound 8c

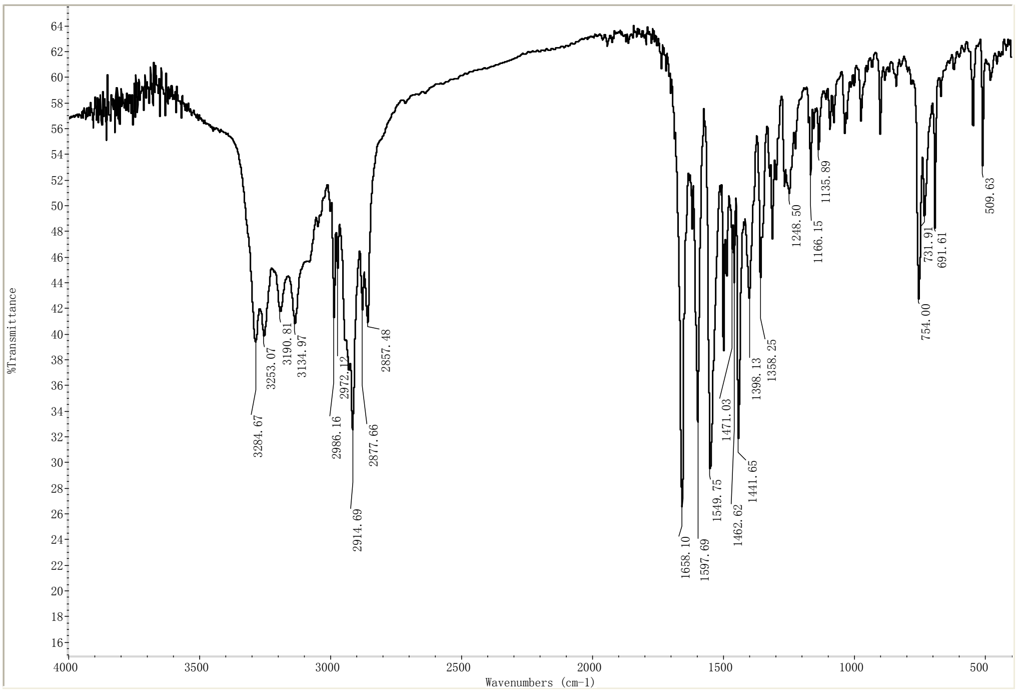

Compound 8d

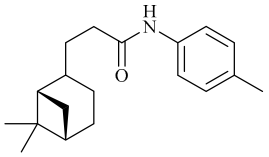

3-((1S,5S)-6,6-dimethylbicyclo[3.1.1]heptan-2-yl)-N-(p-tolyl)propanamide

FTIR of compound 8d

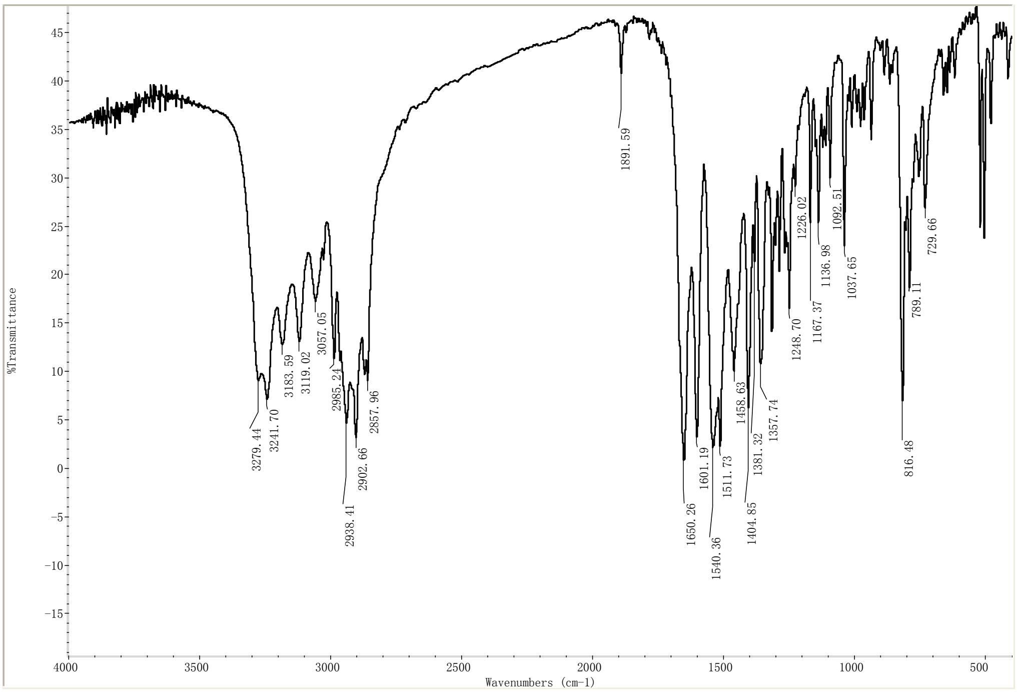

Compound 8e

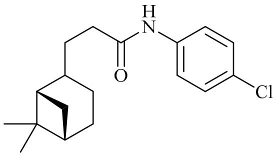

N-(4-chlorophenyl)-3-((1S,5S)-6,6-dimethylbicyclo[3.1.1]heptan-2-yl)propanamide

FTIR of compound 8e

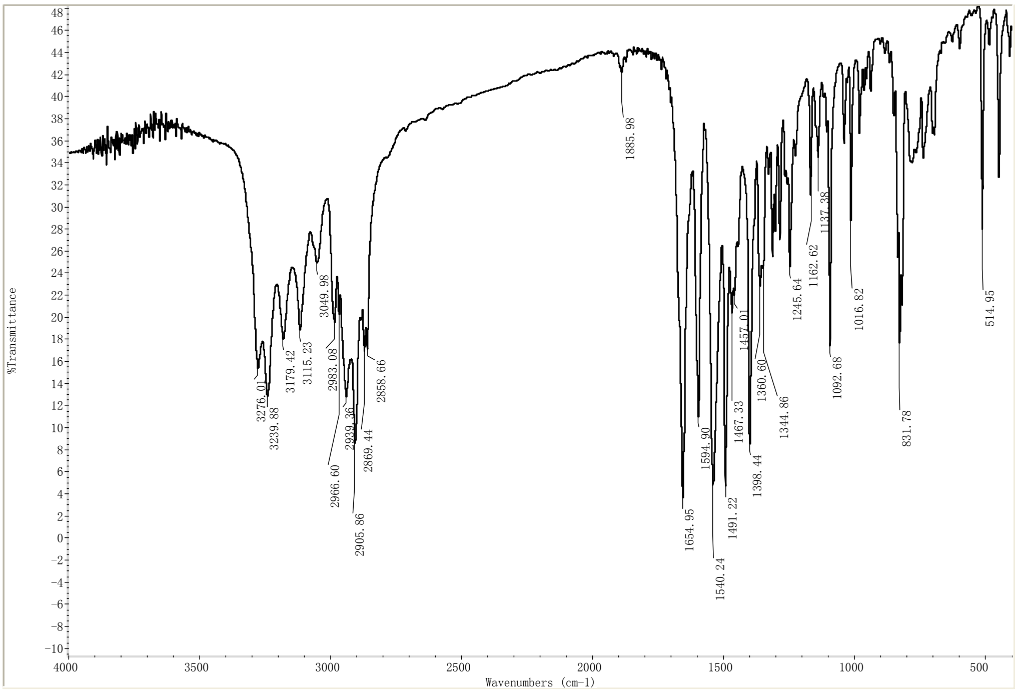

Compound 8f

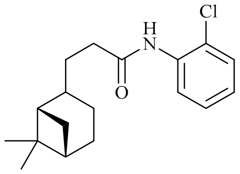

N-(2-chlorophenyl)-3-((1S,5S)-6,6-dimethylbicyclo[3.1.1]heptan-2-yl)propanamide

FTIR of compound 8f

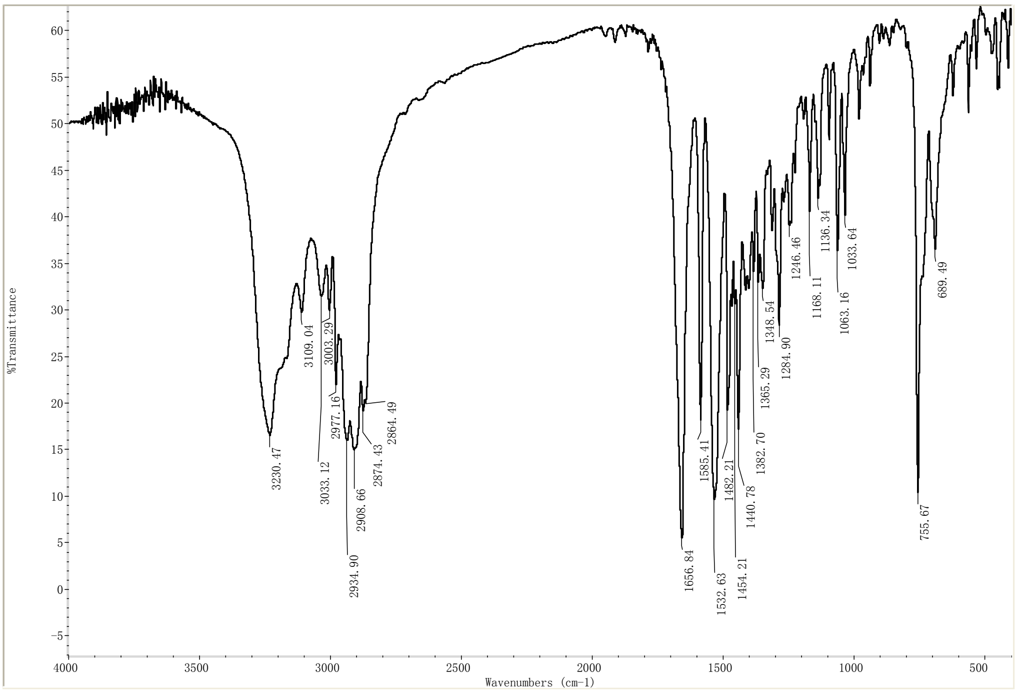

Compound 8g

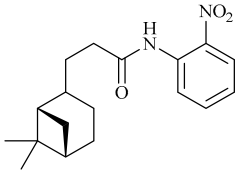

3-((1S,5S)-6,6-dimethylbicyclo[3.1.1]heptan-2-yl)-N-(2-nitrophenyl)propanamide

FTIR of compound 8g

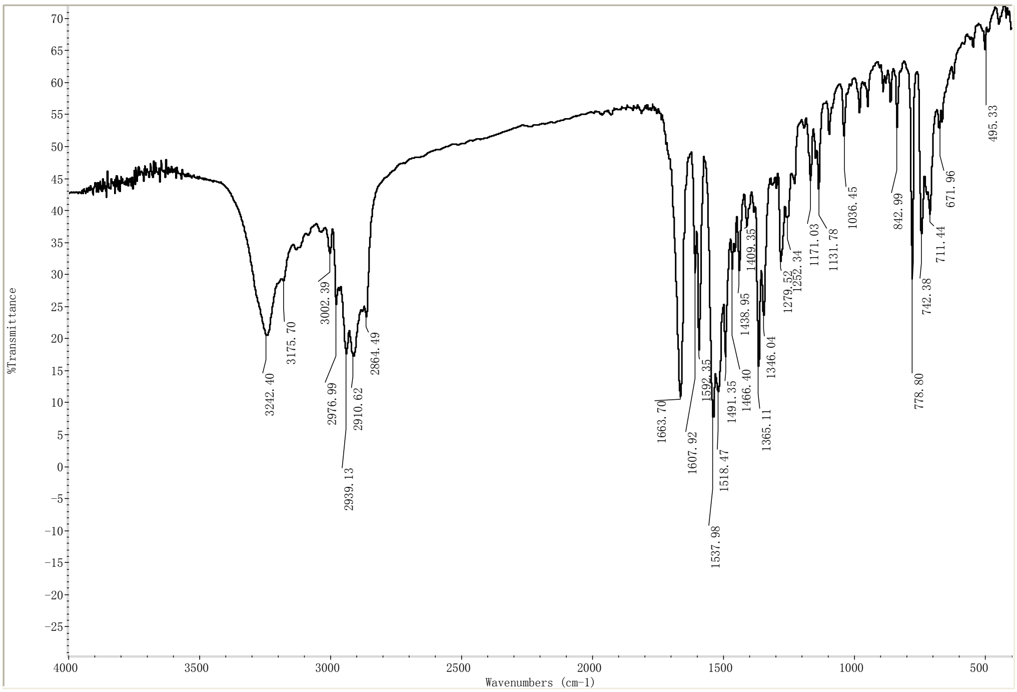

NMR spectrums of compound 8a-8g

^{1}H NMR of compound 8a:

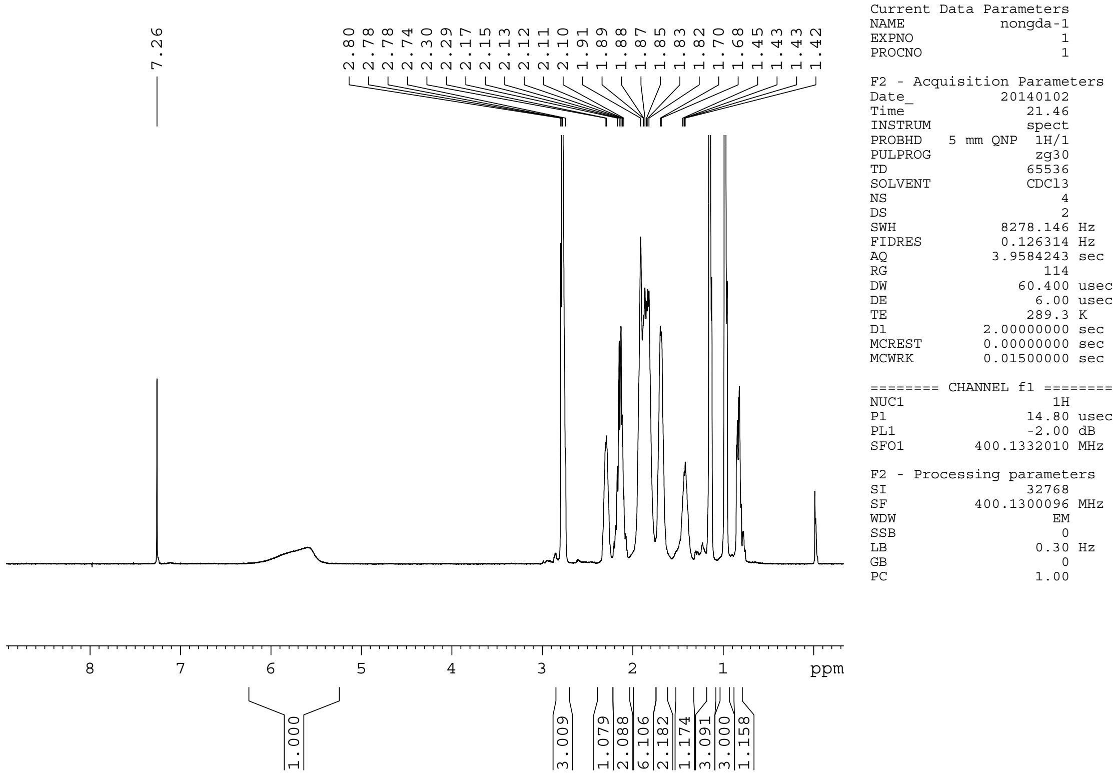

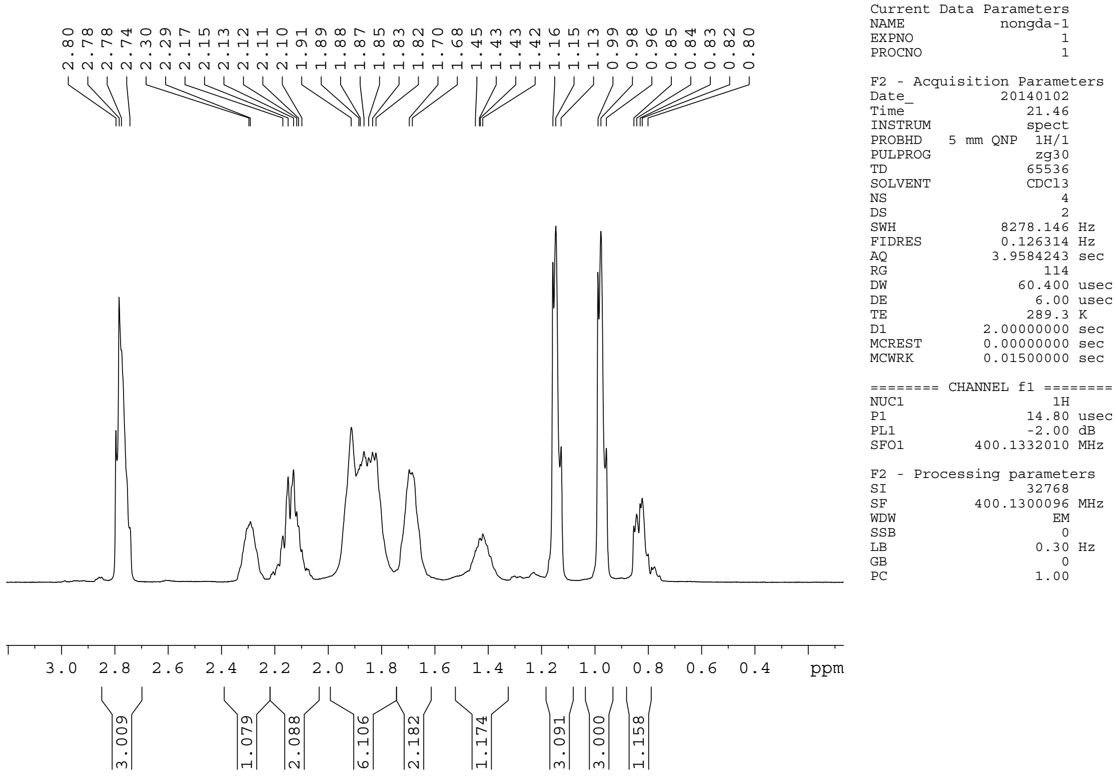

^{13}C NMR of compound 8a:

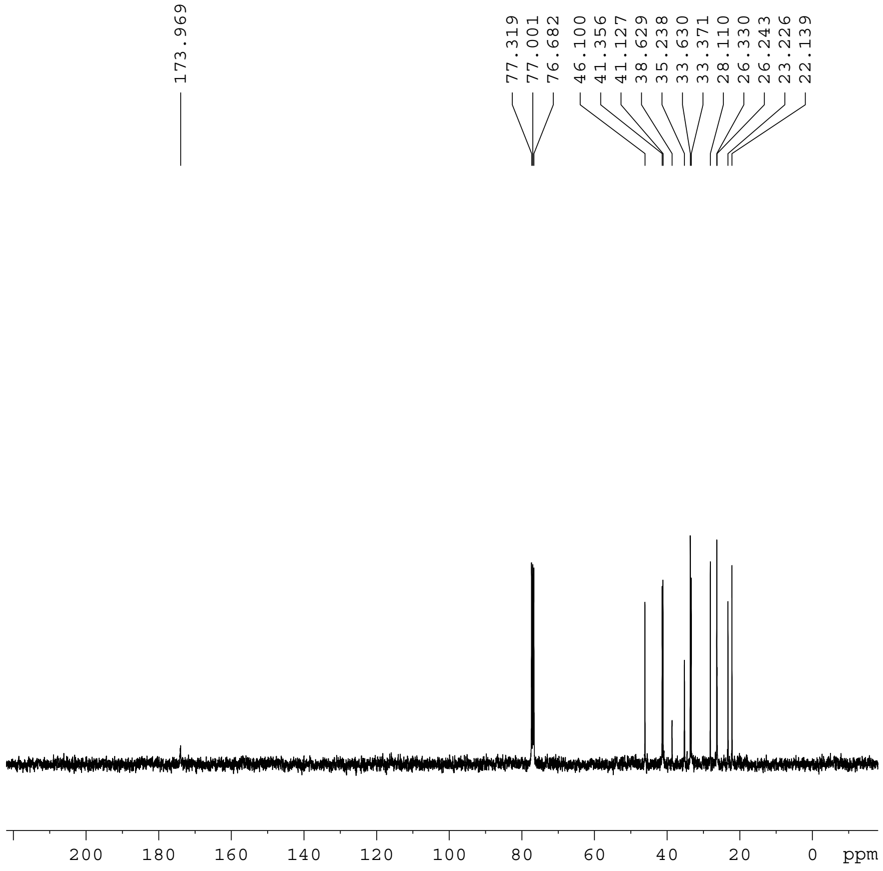


```
Current Data Parameters
NAME           nongda-1
EXPNO                 2
PROCNO                1

F2 - Acquisition Parameters
Date_          20140102
Time              21.47
INSTRUM           spect
PROBHD   5 mm QNP  1H/1
PULPROG         zgdc30
TD               32768
SOLVENT           DMSO
NS                 120
DS                   0
SWH          24154.590 Hz
FIDRES        0.737140 Hz
AQ           0.6783476 sec
RG                 362
DW              20.700 usec
DE                6.00 usec
TE               289.9 K
D1          1.00000000 sec
d11         0.03000000 sec
MCREST      0.00000000 sec
MCWRK       0.01500000 sec

======== CHANNEL f1 ========
NUC1               13C
P1               12.00 usec
PL1              -3.00 dB
SFO1       100.6230315 MHz

======== CHANNEL f2 ========
CPDPRG2        waltz16
NUC2                1H
PCPD2            80.00 usec
PL2              -4.00 dB
PL12             12.12 dB
SFO2       400.1313965 MHz

F2 - Processing parameters
SI              131072
SF         100.6127747 MHz
WDW                 EM
SSB                  0
LB                3.00 Hz
GB                   0
PC                0.50
```

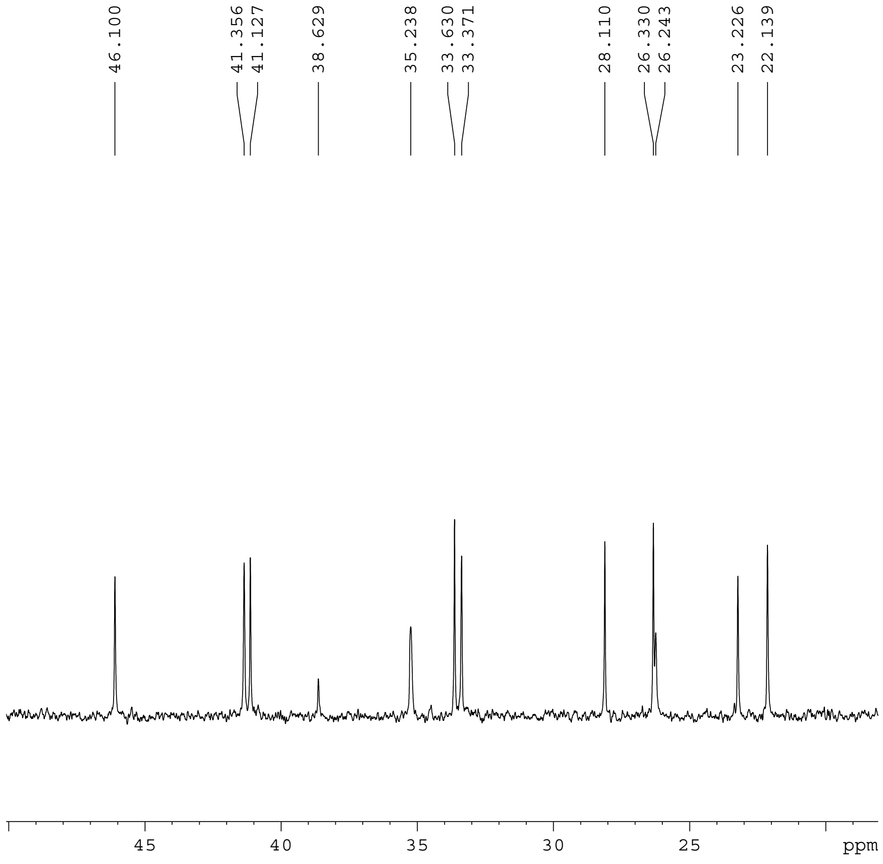

Current Data Parameters
NAME nongda-1
EXPNO 2
PROCNO 1

F2 - Acquisition Parameters
Date_ 20140102
Time 21.47
INSTRUM spect
PROBHD 5 mm QNP 1H/1
PULPROG zgdc30
TD 32768
SOLVENT DMSO
NS 120
DS 0
SWH 24154.590 Hz
FIDRES 0.737140 Hz
AQ 0.6783476 sec
RG 362
DW 20.700 usec
DE 6.00 usec
TE 289.9 K
D1 1.00000000 sec
d11 0.03000000 sec
MCREST 0.00000000 sec
MCWRK 0.01500000 sec

======== CHANNEL f1 ========
NUC1 13C
P1 12.00 usec
PL1 -3.00 dB
SFO1 100.6230315 MHz

======== CHANNEL f2 ========
CPDPRG2 waltz16
NUC2 1H
PCPD2 80.00 usec
PL2 -4.00 dB
PL12 12.12 dB
SFO2 400.1313965 MHz

F2 - Processing parameters
SI 131072
SF 100.6127747 MHz
WDW EM
SSB 0
LB 3.00 Hz
GB 0
PC 0.50

^{1}H NMR of compound 8b:

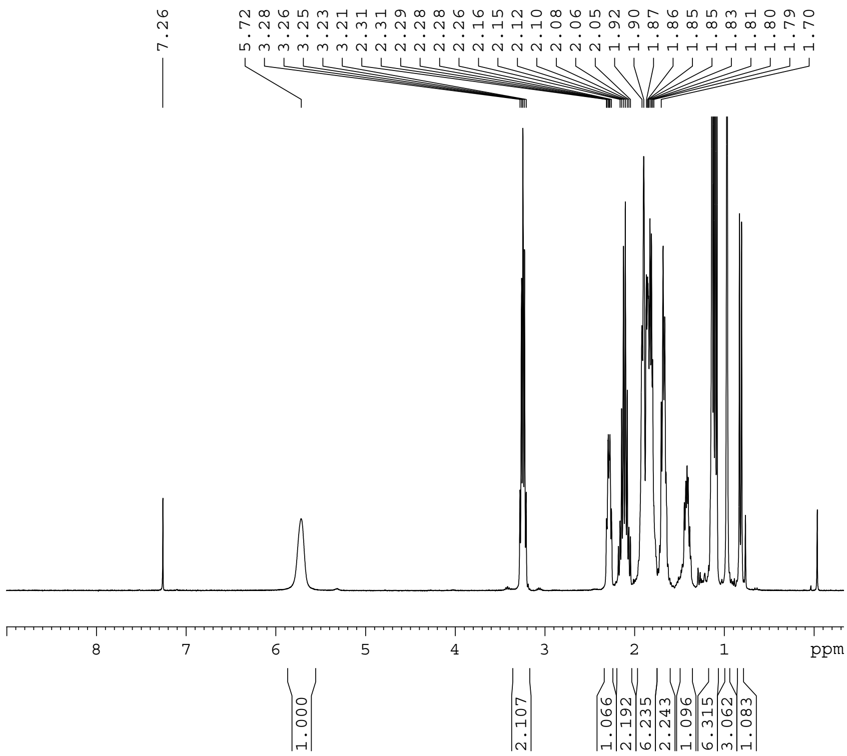


```
Current Data Parameters
NAME            nongda-2
EXPNO                  1
PROCNO                 1

F2 - Acquisition Parameters
Date_           20140105
Time               14.48
INSTRUM            spect
PROBHD   5 mm QNP  1H/1
PULPROG             zg30
TD                 65536
SOLVENT            CDCl3
NS                     4
DS                     2
SWH             8278.146 Hz
FIDRES          0.126314 Hz
AQ             3.9584243 sec
RG                    57
DW                60.400 usec
DE                  6.00 usec
TE                 288.2 K
D1            2.00000000 sec
MCREST        0.00000000 sec
MCWRK         0.01500000 sec

======== CHANNEL f1 ========
NUC1                  1H
P1                 14.80 usec
PL1                -2.00 dB
SFO1         400.1332010 MHz

F2 - Processing parameters
SI                 32768
SF           400.1300096 MHz
WDW                   EM
SSB                    0
LB                  0.30 Hz
GB                     0
PC                  1.00
```

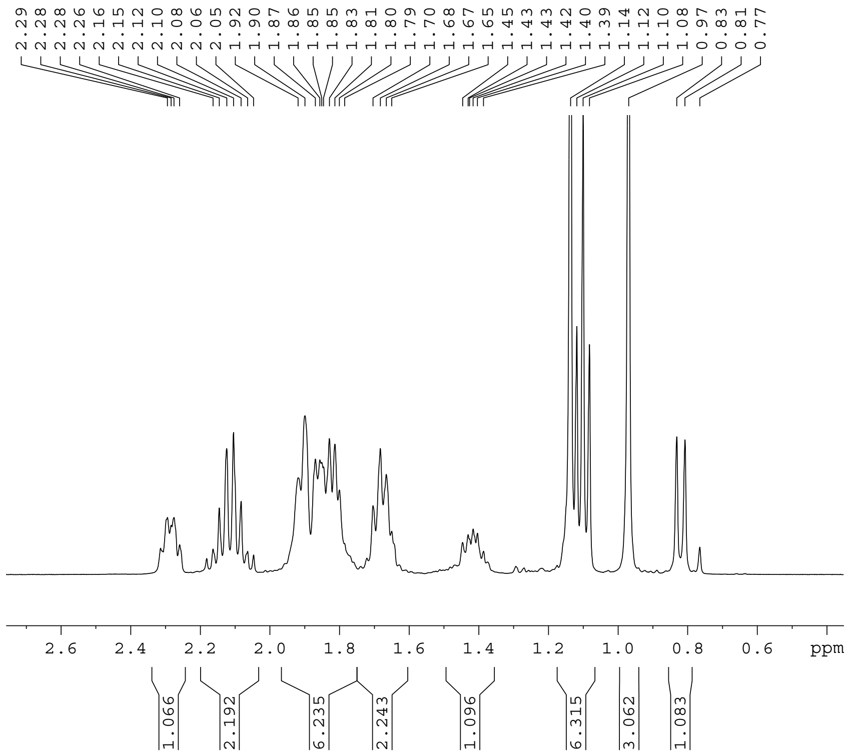


```
Current Data Parameters
NAME            nongda-2
EXPNO                  1
PROCNO                 1

F2 - Acquisition Parameters
Date_           20140105
Time               14.48
INSTRUM            spect
PROBHD   5 mm QNP  1H/1
PULPROG             zg30
TD                 65536
SOLVENT            CDCl3
NS                     4
DS                     2
SWH             8278.146 Hz
FIDRES          0.126314 Hz
AQ             3.9584243 sec
RG                    57
DW                60.400 usec
DE                  6.00 usec
TE                 288.2 K
D1            2.00000000 sec
MCREST        0.00000000 sec
MCWRK         0.01500000 sec

======== CHANNEL f1 ========
NUC1                  1H
P1                 14.80 usec
PL1                -2.00 dB
SFO1         400.1332010 MHz

F2 - Processing parameters
SI                 32768
SF           400.1300096 MHz
WDW                   EM
SSB                    0
LB                  0.30 Hz
GB                     0
PC                  1.00
```

^{13}C NMR of compound 8b:

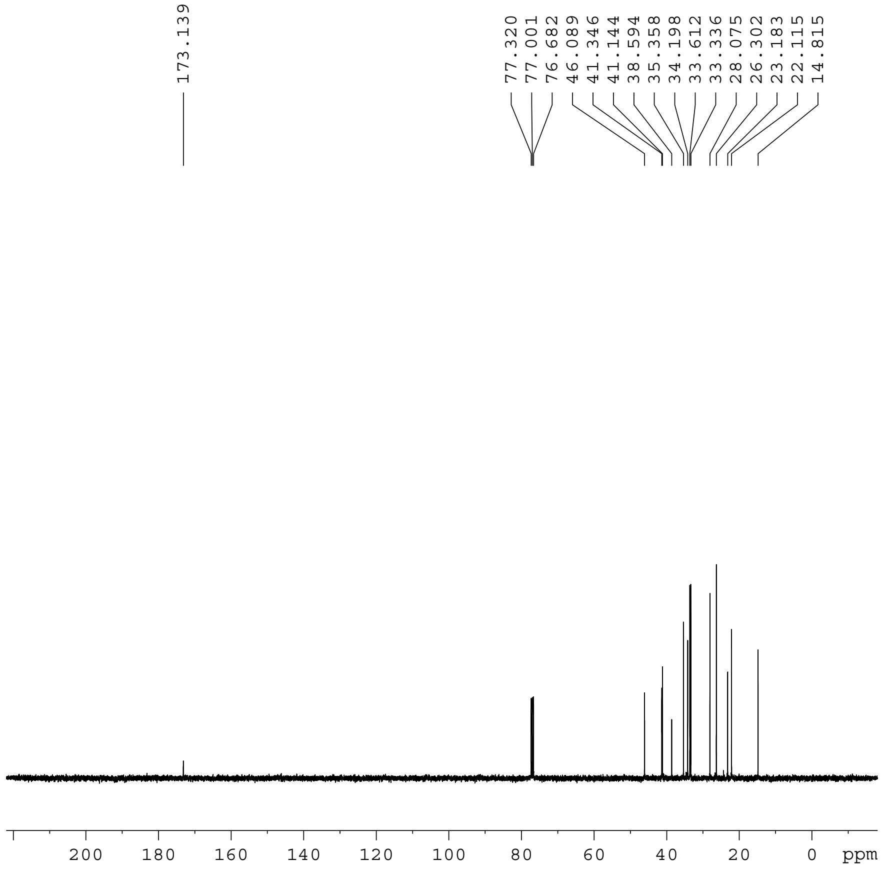


```
Current Data Parameters
NAME            nongda-2
EXPNO                  2
PROCNO                 1

F2 - Acquisition Parameters
Date_           20140105
Time               14.54
INSTRUM            spect
PROBHD   5 mm QNP  1H/1
PULPROG           zgdc30
TD                 32768
SOLVENT          Acetone
NS                   147
DS                     0
SWH           24154.590 Hz
FIDRES         0.737140 Hz
AQ            0.6783476 sec
RG                   362
DW               20.700 usec
DE                 6.00 usec
TE                289.7 K
D1           1.00000000 sec
d11          0.03000000 sec
MCREST       0.00000000 sec
MCWRK        0.01500000 sec

======== CHANNEL f1 ========
NUC1                 13C
P1                 12.00 usec
PL1                -3.00 dB
SFO1         100.6230315 MHz

======== CHANNEL f2 ========
CPDPRG2          waltz16
NUC2                  1H
PCPD2              80.00 usec
PL2                -4.00 dB
PL12               12.12 dB
SFO2         400.1313965 MHz

F2 - Processing parameters
SI                131072
SF           100.6127777 MHz
WDW                   EM
SSB                    0
LB                  1.00 Hz
GB                     0
PC                  0.50
```

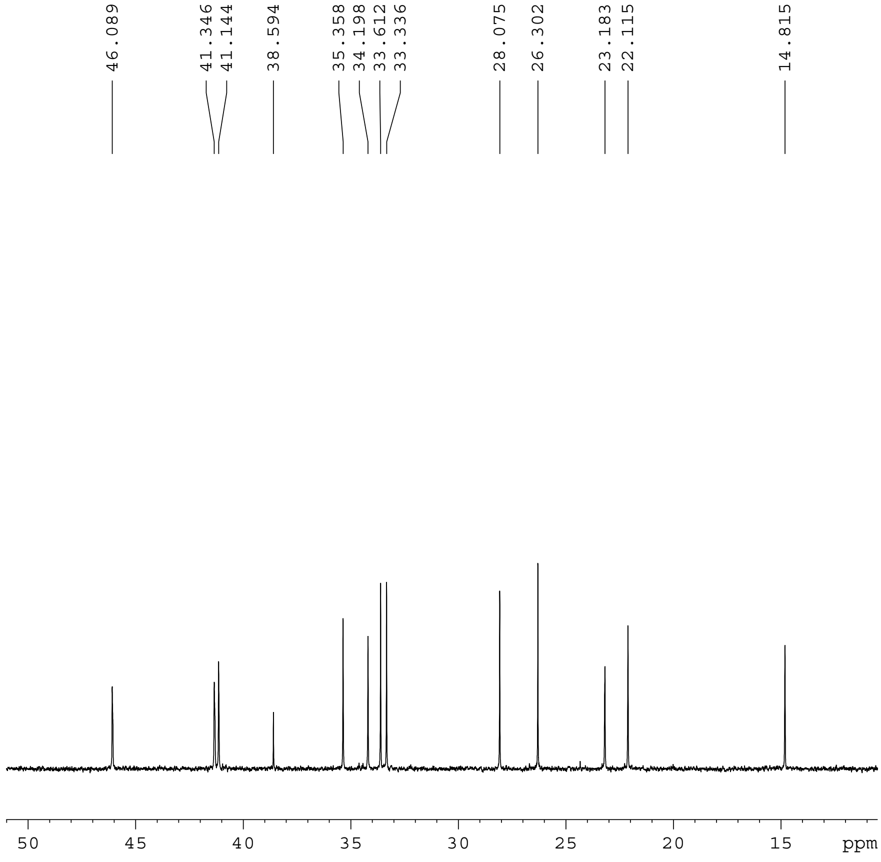


```
Current Data Parameters
NAME            nongda-2
EXPNO                  2
PROCNO                 1

F2 - Acquisition Parameters
Date_           20140105
Time               14.54
INSTRUM            spect
PROBHD   5 mm QNP  1H/1
PULPROG           zgdc30
TD                 32768
SOLVENT          Acetone
NS                   147
DS                     0
SWH           24154.590 Hz
FIDRES         0.737140 Hz
AQ            0.6783476 sec
RG                   362
DW               20.700 usec
DE                 6.00 usec
TE                289.7 K
D1           1.00000000 sec
d11          0.03000000 sec
MCREST       0.00000000 sec
MCWRK        0.01500000 sec

======== CHANNEL f1 ========
NUC1                 13C
P1                 12.00 usec
PL1                -3.00 dB
SFO1         100.6230315 MHz

======== CHANNEL f2 ========
CPDPRG2          waltz16
NUC2                  1H
PCPD2              80.00 usec
PL2                -4.00 dB
PL12               12.12 dB
SFO2         400.1313965 MHz

F2 - Processing parameters
SI                131072
SF           100.6127777 MHz
WDW                   EM
SSB                    0
LB                  1.00 Hz
GB                     0
PC                  0.50
```

^{1}H NMR of compound 8c:

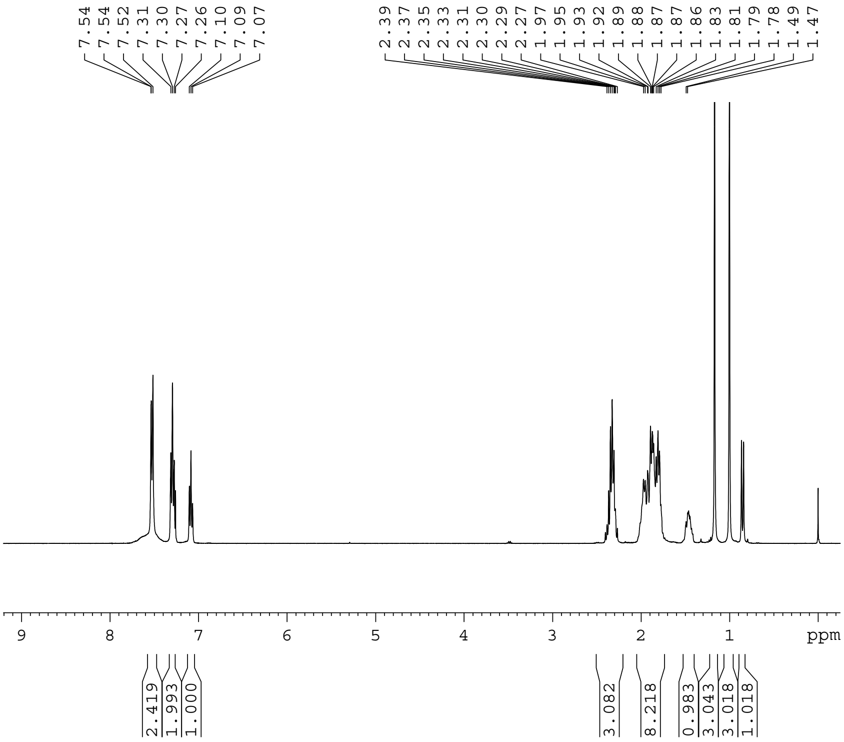

Current Data Parameters
NAME nongda-3
EXPNO 1
PROCNO 1

F2 - Acquisition Parameters
Date_ 20140103
Time 14.04
INSTRUM spect
PROBHD 5 mm QNP 1H/1
PULPROG zg30
TD 65536
SOLVENT Pyr
NS 4
DS 2
SWH 8278.146 Hz
FIDRES 0.126314 Hz
AQ 3.9584243 sec
RG 57
DW 60.400 usec
DE 6.00 usec
TE 288.1 K
D1 2.00000000 sec
MCREST 0.00000000 sec
MCWRK 0.01500000 sec

======== CHANNEL f1 ========
NUC1 1H
P1 14.80 usec
PL1 -2.00 dB
SFO1 400.1332010 MHz

F2 - Processing parameters
SI 32768
SF 400.1300093 MHz
WDW EM
SSB 0
LB 0.30 Hz
GB 0
PC 1.00

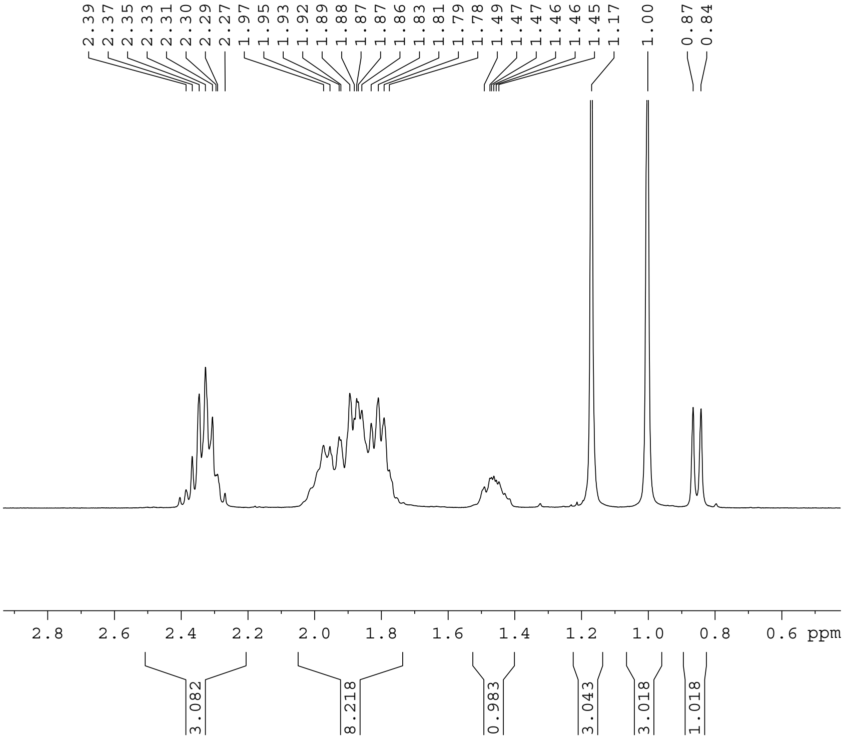

Current Data Parameters
NAME nongda-3
EXPNO 1
PROCNO 1

F2 - Acquisition Parameters
Date_ 20140103
Time 14.04
INSTRUM spect
PROBHD 5 mm QNP 1H/1
PULPROG zg30
TD 65536
SOLVENT Pyr
NS 4
DS 2
SWH 8278.146 Hz
FIDRES 0.126314 Hz
AQ 3.9584243 sec
RG 57
DW 60.400 usec
DE 6.00 usec
TE 288.1 K
D1 2.00000000 sec
MCREST 0.00000000 sec
MCWRK 0.01500000 sec

======== CHANNEL f1 ========
NUC1 1H
P1 14.80 usec
PL1 -2.00 dB
SFO1 400.1332010 MHz

F2 - Processing parameters
SI 32768
SF 400.1300093 MHz
WDW EM
SSB 0
LB 0.30 Hz
GB 0
PC 1.00

^{13}C NMR of compound 8c:

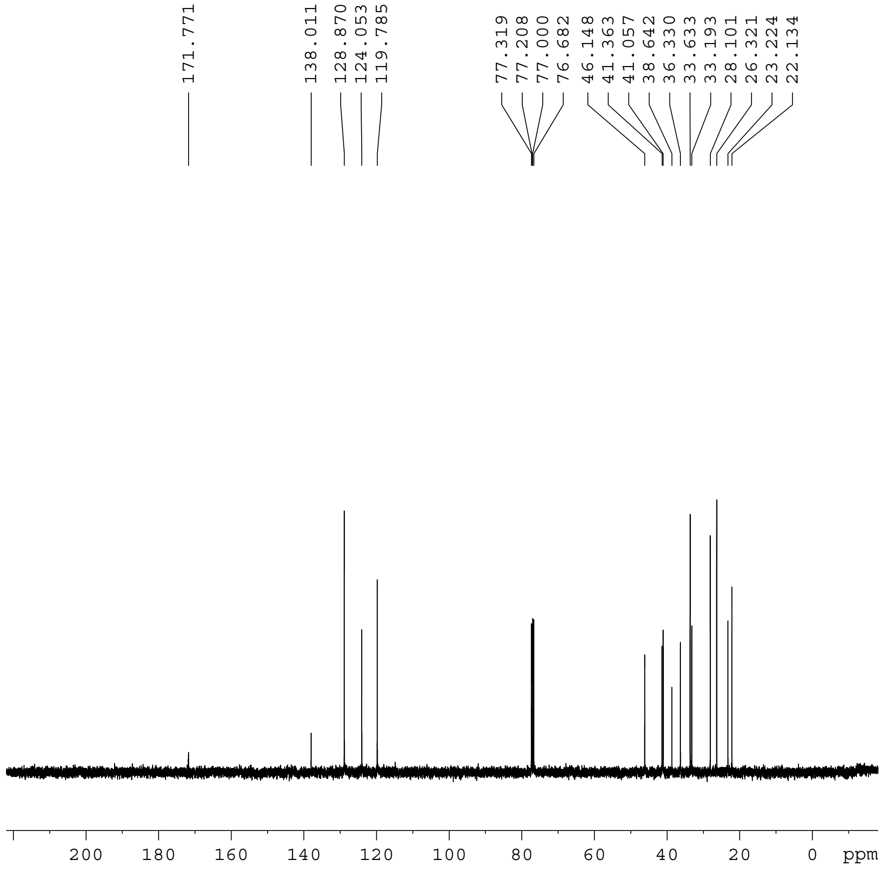


```
Current Data Parameters
NAME          nongda-3-1
EXPNO                  2
PROCNO                 1

F2 - Acquisition Parameters
Date_           20140106
Time               15.53
INSTRUM            spect
PROBHD   5 mm QNP  1H/1
PULPROG           zgdc30
TD                 32768
SOLVENT          Acetone
NS                   102
DS                     0
SWH            24154.590 Hz
FIDRES          0.737140 Hz
AQ             0.6783476 sec
RG                   362
DW                20.700 usec
DE                  6.00 usec
TE                 290.5 K
D1            1.00000000 sec
d11           0.03000000 sec
MCREST        0.00000000 sec
MCWRK         0.01500000 sec

======== CHANNEL f1 ========
NUC1                 13C
P1                 12.00 usec
PL1                -3.00 dB
SFO1         100.6230315 MHz

======== CHANNEL f2 ========
CPDPRG2          waltz16
NUC2                  1H
PCPD2              80.00 usec
PL2                -4.00 dB
PL12               12.12 dB
SFO2         400.1313965 MHz

F2 - Processing parameters
SI                131072
SF           100.6127769 MHz
WDW                   EM
SSB                    0
LB                  1.00 Hz
GB                     0
PC                  0.50
```

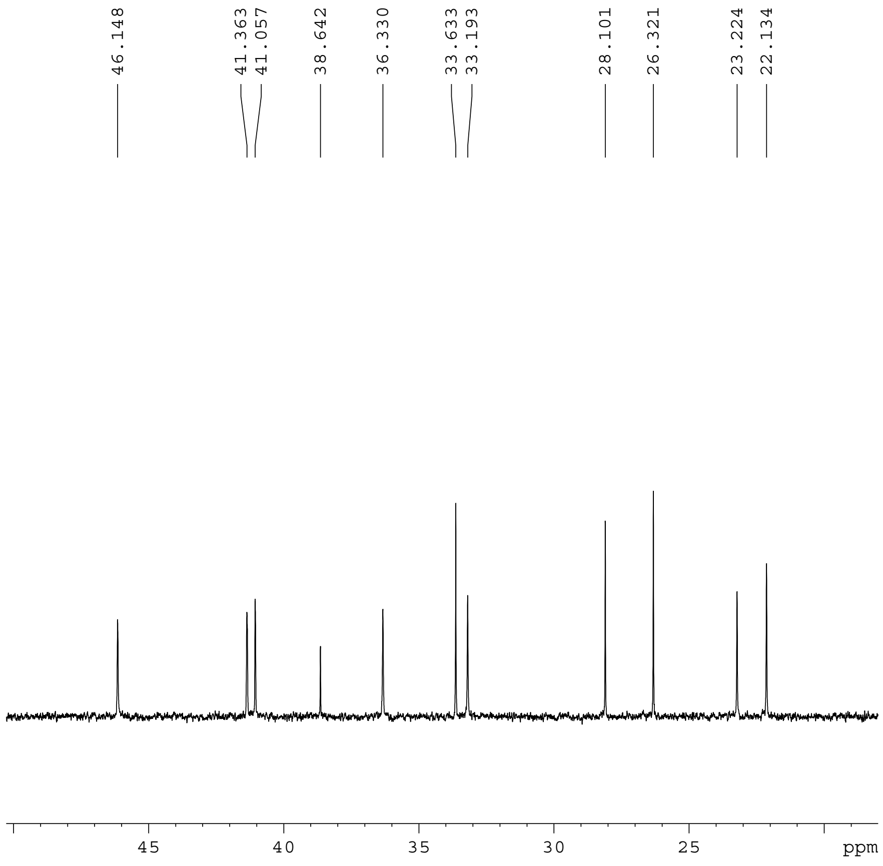


```
Current Data Parameters
NAME          nongda-3-1
EXPNO                  2
PROCNO                 1

F2 - Acquisition Parameters
Date_           20140106
Time               15.53
INSTRUM            spect
PROBHD   5 mm QNP  1H/1
PULPROG           zgdc30
TD                 32768
SOLVENT          Acetone
NS                   102
DS                     0
SWH            24154.590 Hz
FIDRES          0.737140 Hz
AQ             0.6783476 sec
RG                   362
DW                20.700 usec
DE                  6.00 usec
TE                 290.5 K
D1            1.00000000 sec
d11           0.03000000 sec
MCREST        0.00000000 sec
MCWRK         0.01500000 sec

======== CHANNEL f1 ========
NUC1                 13C
P1                 12.00 usec
PL1                -3.00 dB
SFO1         100.6230315 MHz

======== CHANNEL f2 ========
CPDPRG2          waltz16
NUC2                  1H
PCPD2              80.00 usec
PL2                -4.00 dB
PL12               12.12 dB
SFO2         400.1313965 MHz

F2 - Processing parameters
SI                131072
SF           100.6127769 MHz
WDW                   EM
SSB                    0
LB                  1.00 Hz
GB                     0
PC                  0.50
```

^{1}H NMR of compound 8d:

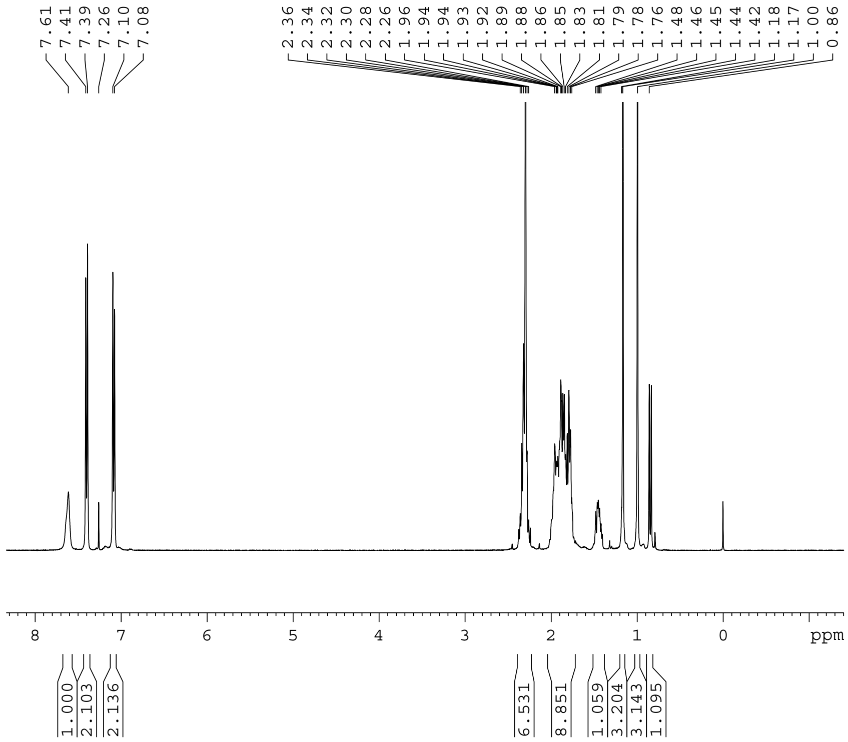

Current Data Parameters
NAME nongda-4
EXPNO 1
PROCNO 1

F2 - Acquisition Parameters
Date_ 20140105
Time 16.22
INSTRUM spect
PROBHD 5 mm QNP 1H/1
PULPROG zg30
TD 65536
SOLVENT CDCl3
NS 4
DS 2
SWH 8278.146 Hz
FIDRES 0.126314 Hz
AQ 3.9584243 sec
RG 57
DW 60.400 usec
DE 6.00 usec
TE 288.7 K
D1 2.00000000 sec
MCREST 0.00000000 sec
MCWRK 0.01500000 sec

======== CHANNEL f1 ========
NUC1 1H
P1 14.80 usec
PL1 -2.00 dB
SFO1 400.1332010 MHz

F2 - Processing parameters
SI 32768
SF 400.1300096 MHz
WDW EM
SSB 0
LB 0.30 Hz
GB 0
PC 1.00

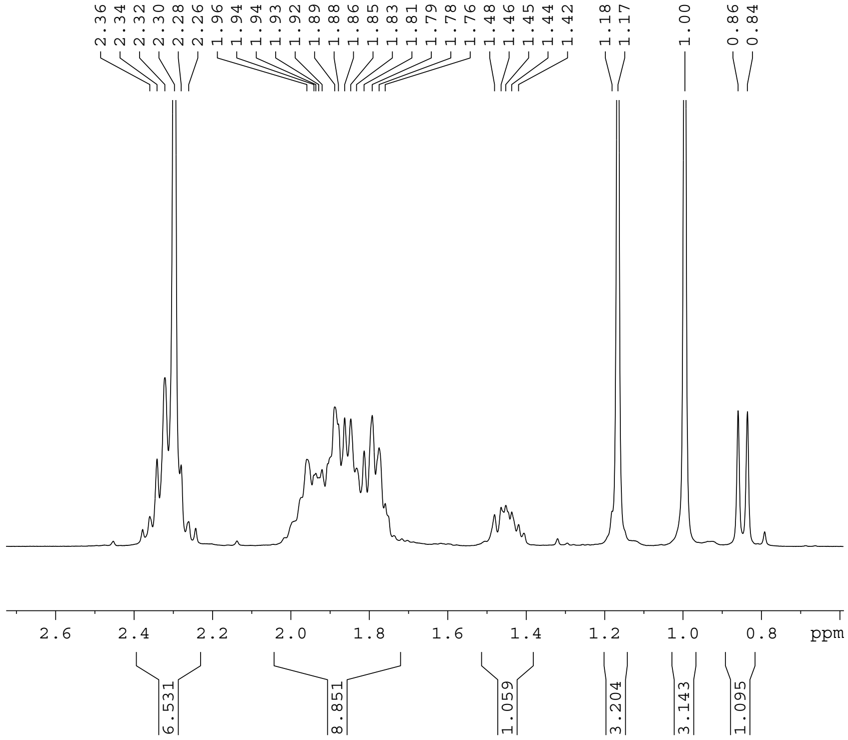

Current Data Parameters
NAME nongda-4
EXPNO 1
PROCNO 1

F2 - Acquisition Parameters
Date_ 20140105
Time 16.22
INSTRUM spect
PROBHD 5 mm QNP 1H/1
PULPROG zg30
TD 65536
SOLVENT CDCl3
NS 4
DS 2
SWH 8278.146 Hz
FIDRES 0.126314 Hz
AQ 3.9584243 sec
RG 57
DW 60.400 usec
DE 6.00 usec
TE 288.7 K
D1 2.00000000 sec
MCREST 0.00000000 sec
MCWRK 0.01500000 sec

======== CHANNEL f1 ========
NUC1 1H
P1 14.80 usec
PL1 -2.00 dB
SFO1 400.1332010 MHz

F2 - Processing parameters
SI 32768
SF 400.1300096 MHz
WDW EM
SSB 0
LB 0.30 Hz
GB 0
PC 1.00

^{13}C NMR of compound 8d:

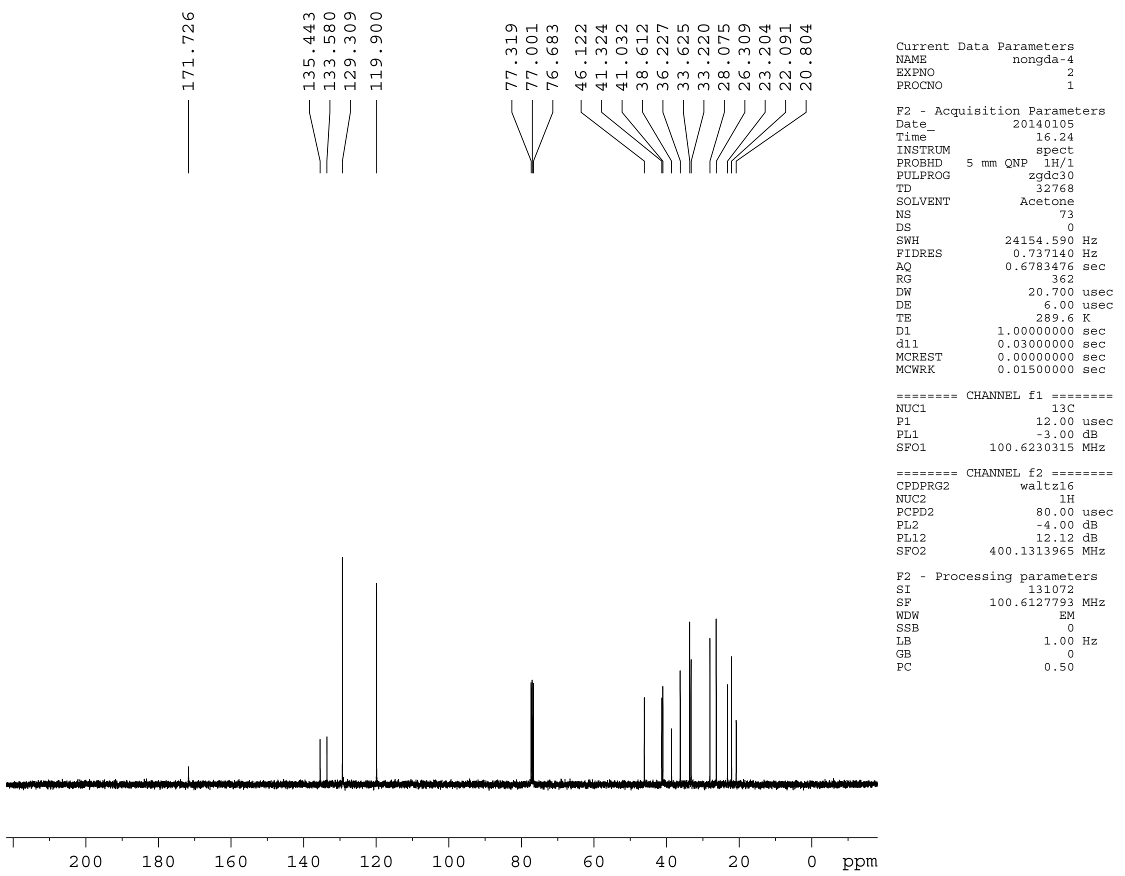

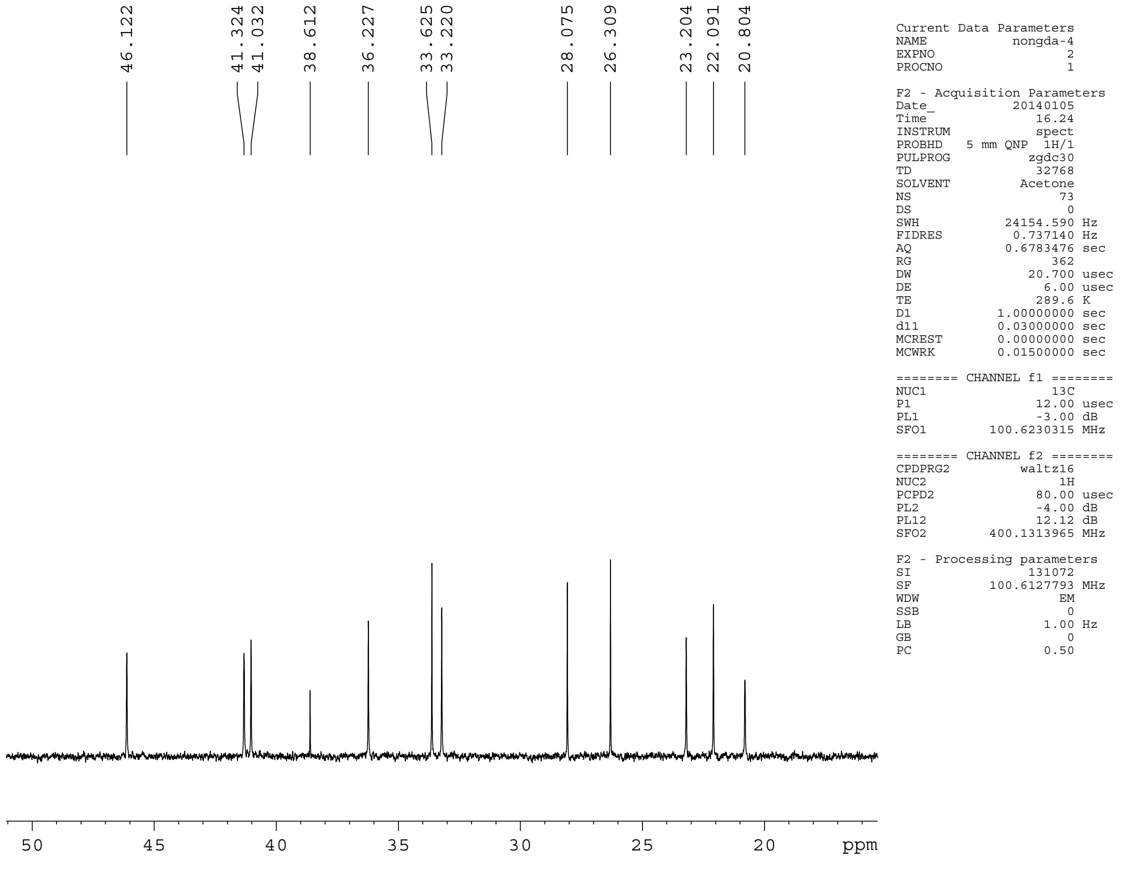

^{1}H NMR of compound 8e:

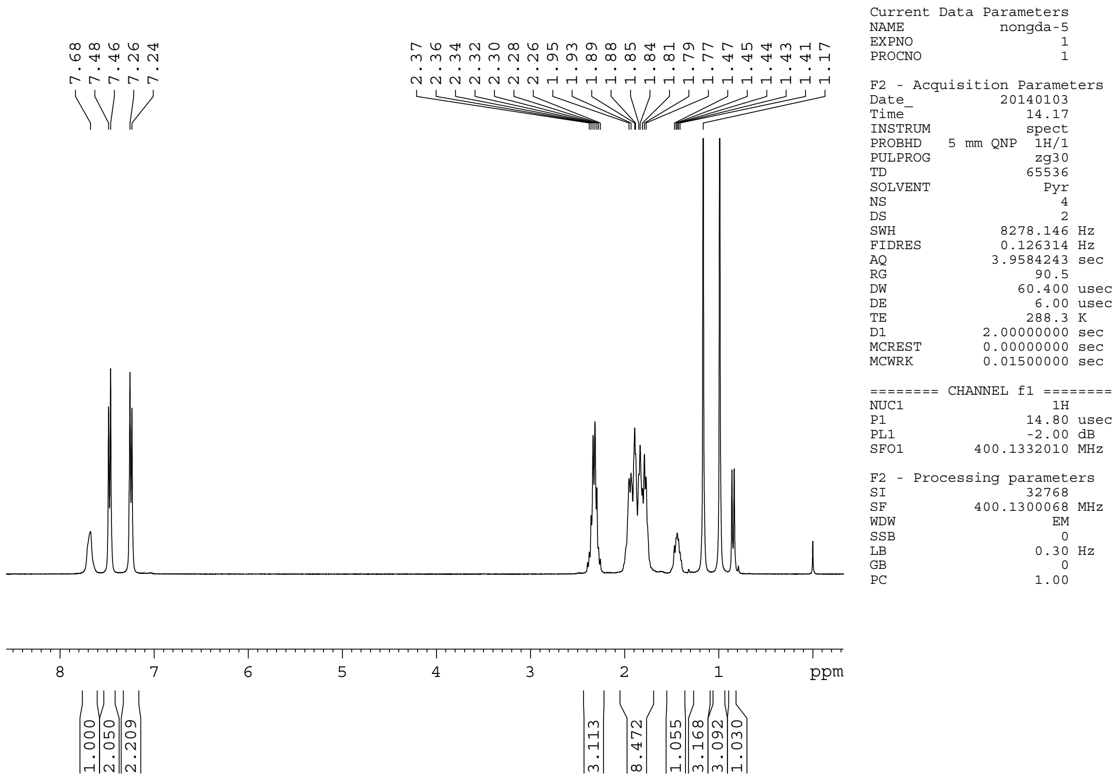

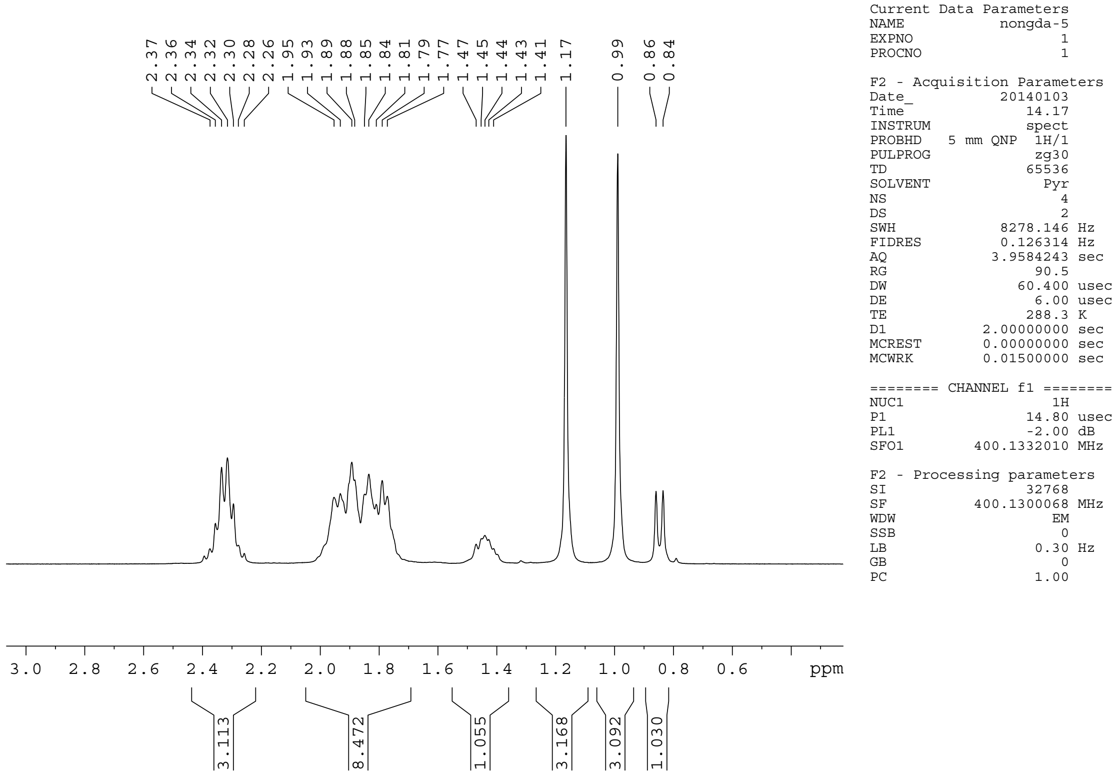

^{13}C NMR of compound 8e:

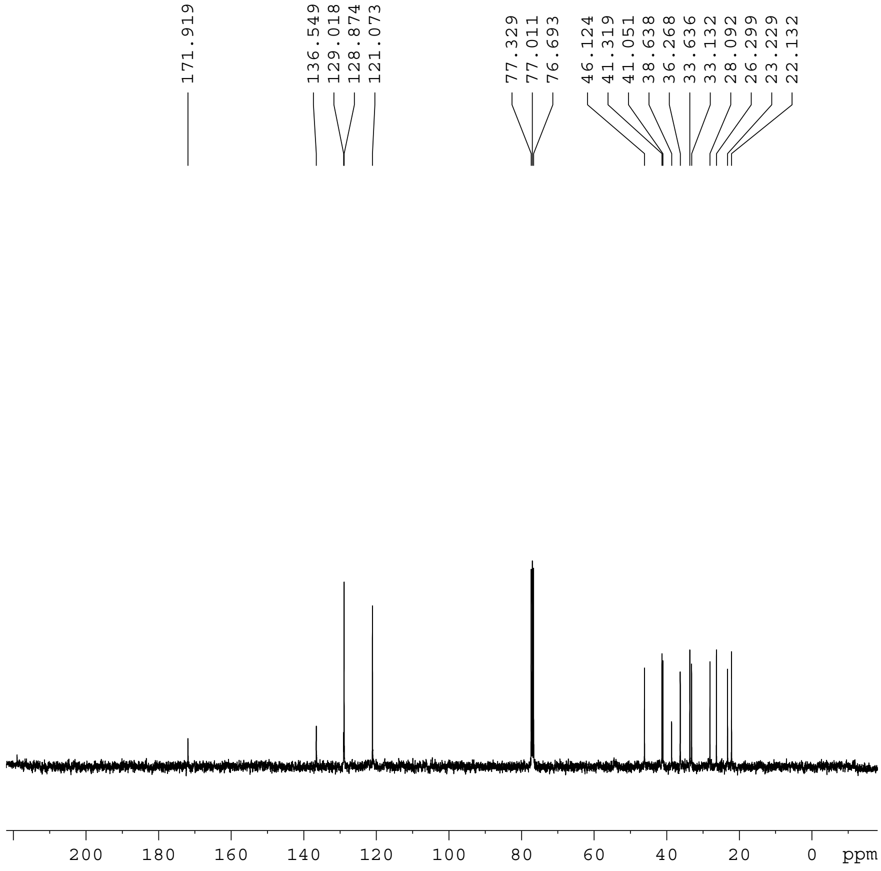

Current Data Parameters
NAME nongda-5
EXPNO 2
PROCNO 1

F2 - Acquisition Parameters
Date_ 20140103
Time 14.21
INSTRUM spect
PROBHD 5 mm QNP 1H/1
PULPROG zgdc30
TD 32768
SOLVENT Acetone
NS 28
DS 0
SWH 24154.590 Hz
FIDRES 0.737140 Hz
AQ 0.6783476 sec
RG 362
DW 20.700 usec
DE 6.00 usec
TE 289.0 K
D1 1.00000000 sec
d11 0.03000000 sec
MCREST 0.00000000 sec
MCWRK 0.01500000 sec

======== CHANNEL f1 ========
NUC1 13C
P1 12.00 usec
PL1 -3.00 dB
SFO1 100.6230315 MHz

======== CHANNEL f2 ========
CPDPRG2 waltz16
NUC2 1H
PCPD2 80.00 usec
PL2 -4.00 dB
PL12 12.12 dB
SFO2 400.1313965 MHz

F2 - Processing parameters
SI 131072
SF 100.6127756 MHz
WDW EM
SSB 0
LB 3.00 Hz
GB 0
PC 0.50

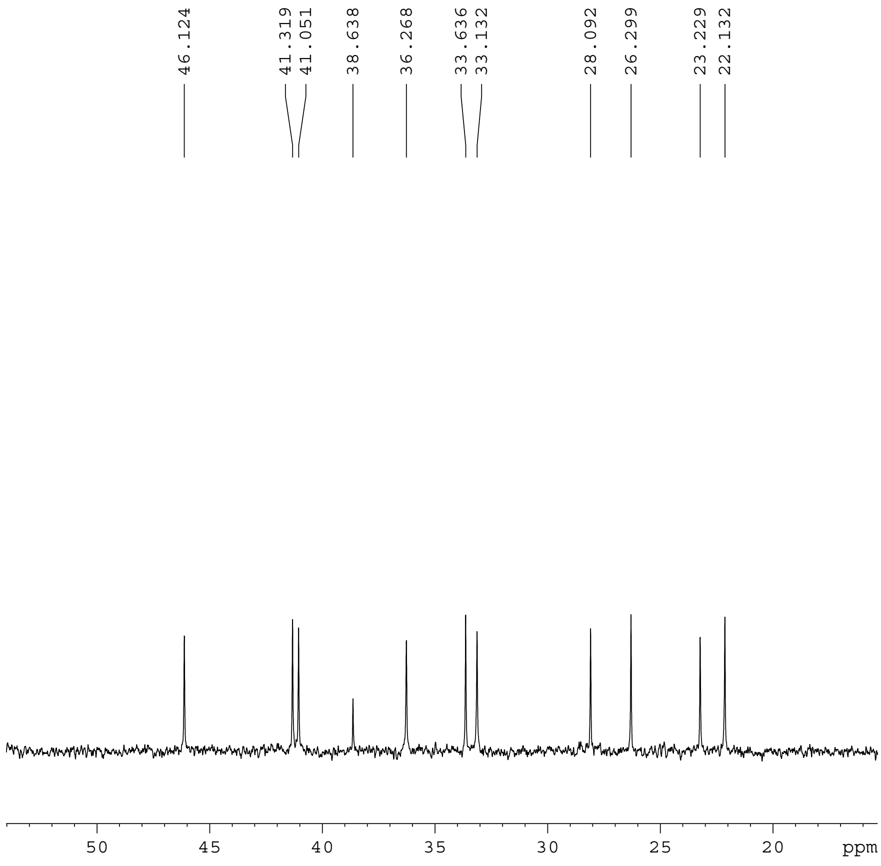

Current Data Parameters
NAME nongda-5
EXPNO 2
PROCNO 1

F2 - Acquisition Parameters
Date_ 20140103
Time 14.21
INSTRUM spect
PROBHD 5 mm QNP 1H/1
PULPROG zgdc30
TD 32768
SOLVENT Acetone
NS 28
DS 0
SWH 24154.590 Hz
FIDRES 0.737140 Hz
AQ 0.6783476 sec
RG 362
DW 20.700 usec
DE 6.00 usec
TE 289.0 K
D1 1.00000000 sec
d11 0.03000000 sec
MCREST 0.00000000 sec
MCWRK 0.01500000 sec

======== CHANNEL f1 ========
NUC1 13C
P1 12.00 usec
PL1 -3.00 dB
SFO1 100.6230315 MHz

======== CHANNEL f2 ========
CPDPRG2 waltz16
NUC2 1H
PCPD2 80.00 usec
PL2 -4.00 dB
PL12 12.12 dB
SFO2 400.1313965 MHz

F2 - Processing parameters
SI 131072
SF 100.6127756 MHz
WDW EM
SSB 0
LB 3.00 Hz
GB 0
PC 0.50

^{1}H NMR of compound 8f:

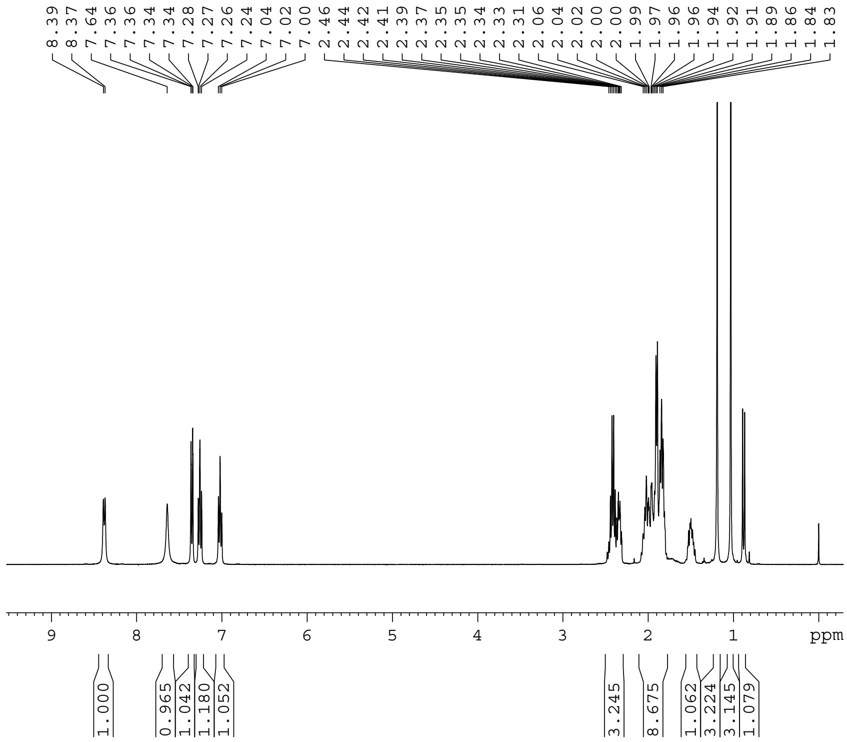


```
Current Data Parameters
NAME            nongda-6
EXPNO                  1
PROCNO                 1

F2 - Acquisition Parameters
Date_           20140103
Time               21.18
INSTRUM            spect
PROBHD   5 mm QNP  1H/1
PULPROG             zg30
TD                 65536
SOLVENT            CDCl3
NS                     4
DS                     2
SWH             8278.146 Hz
FIDRES          0.126314 Hz
AQ             3.9584243 sec
RG                    57
DW                60.400 usec
DE                  6.00 usec
TE                 296.1 K
D1            2.00000000 sec
MCREST        0.00000000 sec
MCWRK         0.01500000 sec

======== CHANNEL f1 ========
NUC1                  1H
P1                 14.80 usec
PL1                -2.00 dB
SFO1         400.1332010 MHz

F2 - Processing parameters
SI                 32768
SF           400.1300085 MHz
WDW                   EM
SSB                    0
LB                  0.30 Hz
GB                     0
PC                  1.00
```

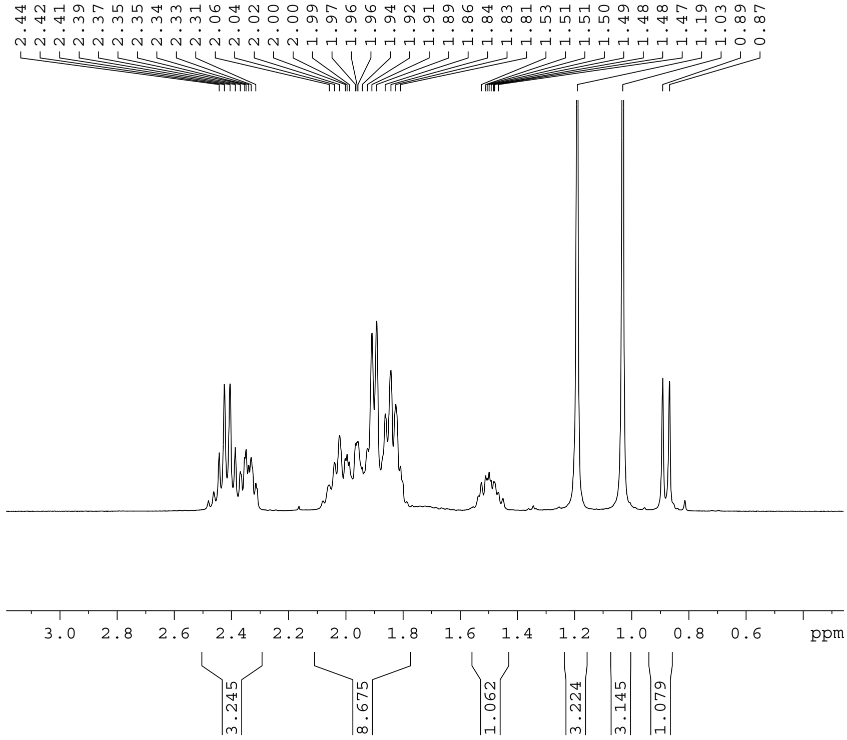


```
Current Data Parameters
NAME            nongda-6
EXPNO                  1
PROCNO                 1

F2 - Acquisition Parameters
Date_           20140103
Time               21.18
INSTRUM            spect
PROBHD   5 mm QNP  1H/1
PULPROG             zg30
TD                 65536
SOLVENT            CDCl3
NS                     4
DS                     2
SWH             8278.146 Hz
FIDRES          0.126314 Hz
AQ             3.9584243 sec
RG                    57
DW                60.400 usec
DE                  6.00 usec
TE                 296.1 K
D1            2.00000000 sec
MCREST        0.00000000 sec
MCWRK         0.01500000 sec

======== CHANNEL f1 ========
NUC1                  1H
P1                 14.80 usec
PL1                -2.00 dB
SFO1         400.1332010 MHz

F2 - Processing parameters
SI                 32768
SF           400.1300085 MHz
WDW                   EM
SSB                    0
LB                  0.30 Hz
GB                     0
PC                  1.00
```

^{13}C NMR of compound 8f:

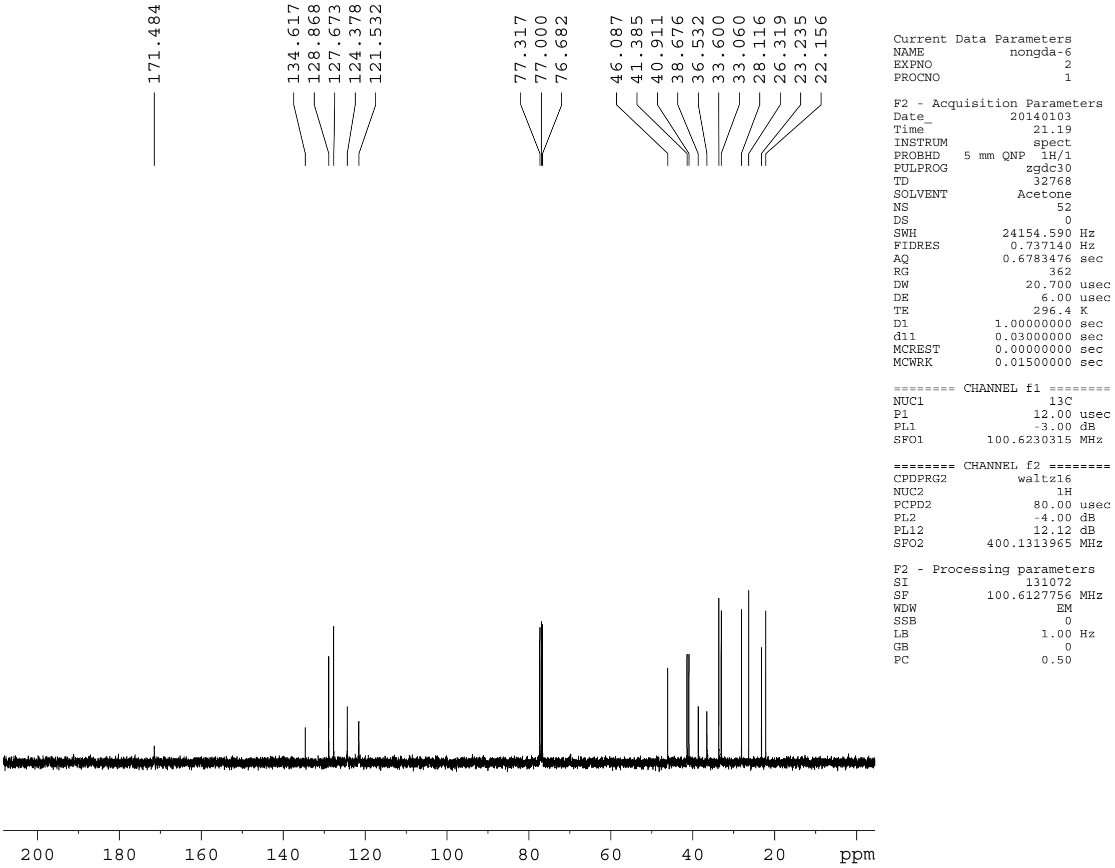

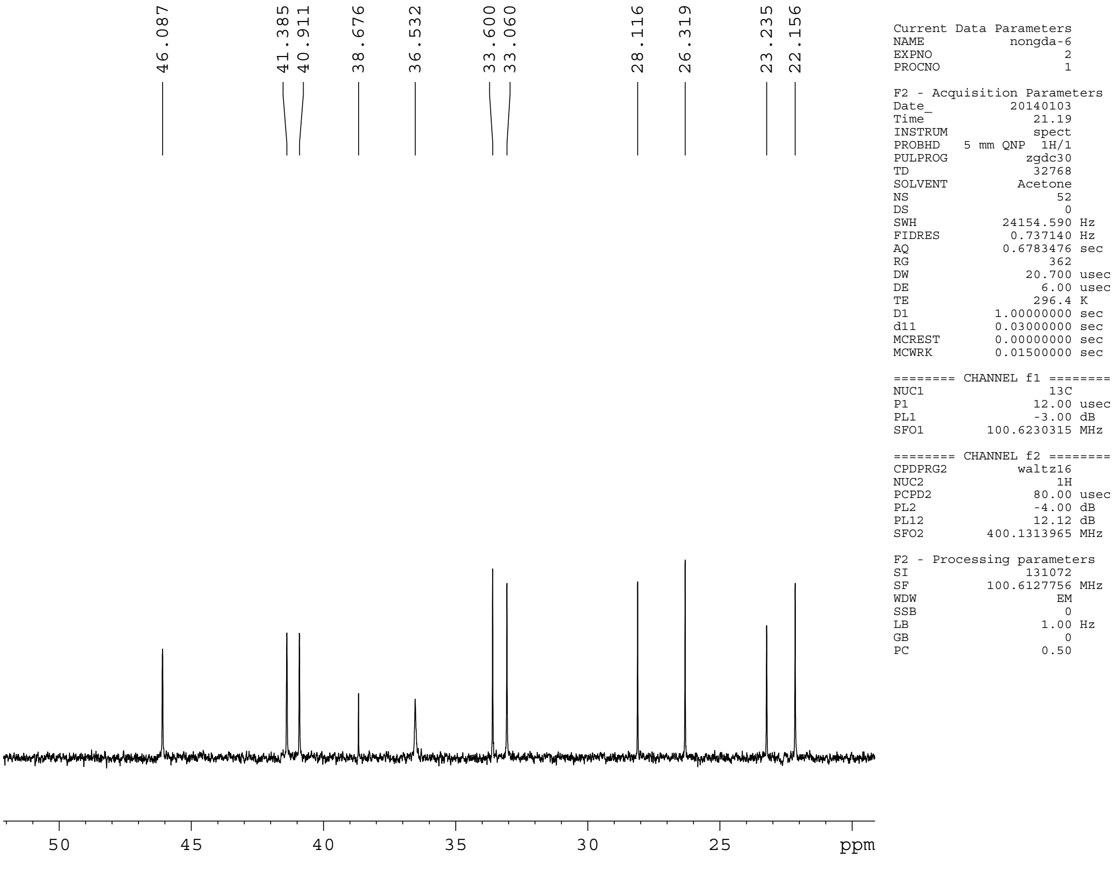

^{1}H NMR of compound 8g:

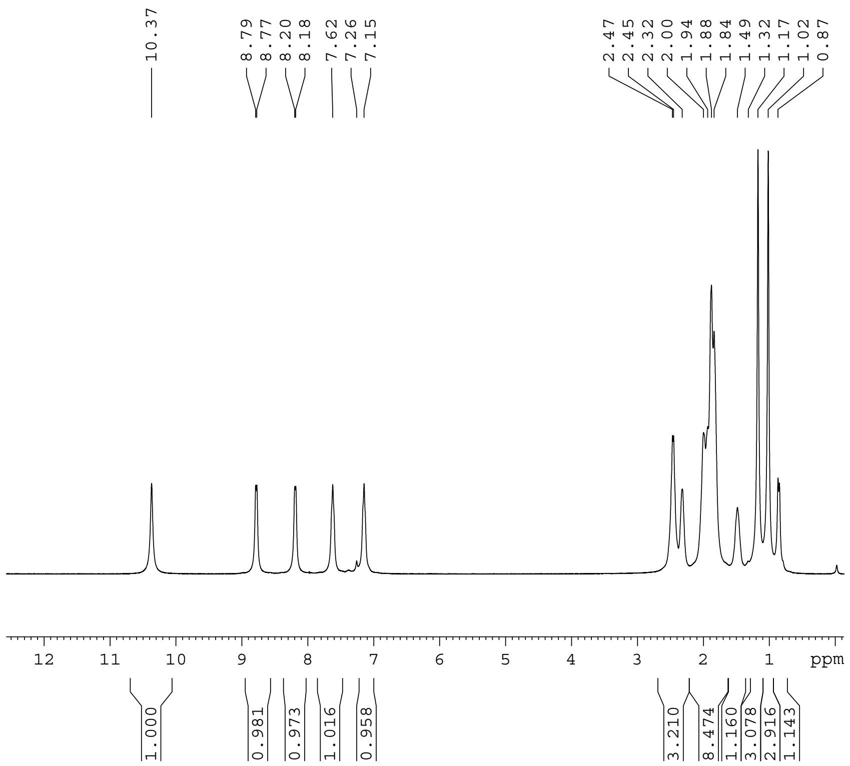

Current Data Parameters
NAME nongda-7
EXPNO 1
PROCNO 1

F2 - Acquisition Parameters
Date_ 20140105
Time 16.41
INSTRUM spect
PROBHD 5 mm QNP 1H/1
PULPROG zg30
TD 65536
SOLVENT CDCl3
NS 4
DS 2
SWH 8278.146 Hz
FIDRES 0.126314 Hz
AQ 3.9584243 sec
RG 50.8
DW 60.400 usec
DE 6.00 usec
TE 288.8 K
D1 2.00000000 sec
MCREST 0.00000000 sec
MCWRK 0.01500000 sec

======== CHANNEL f1 ========
NUC1 1H
P1 14.80 usec
PL1 -2.00 dB
SFO1 400.1332010 MHz

F2 - Processing parameters
SI 32768
SF 400.1300091 MHz
WDW EM
SSB 0
LB 0.30 Hz
GB 0
PC 1.00

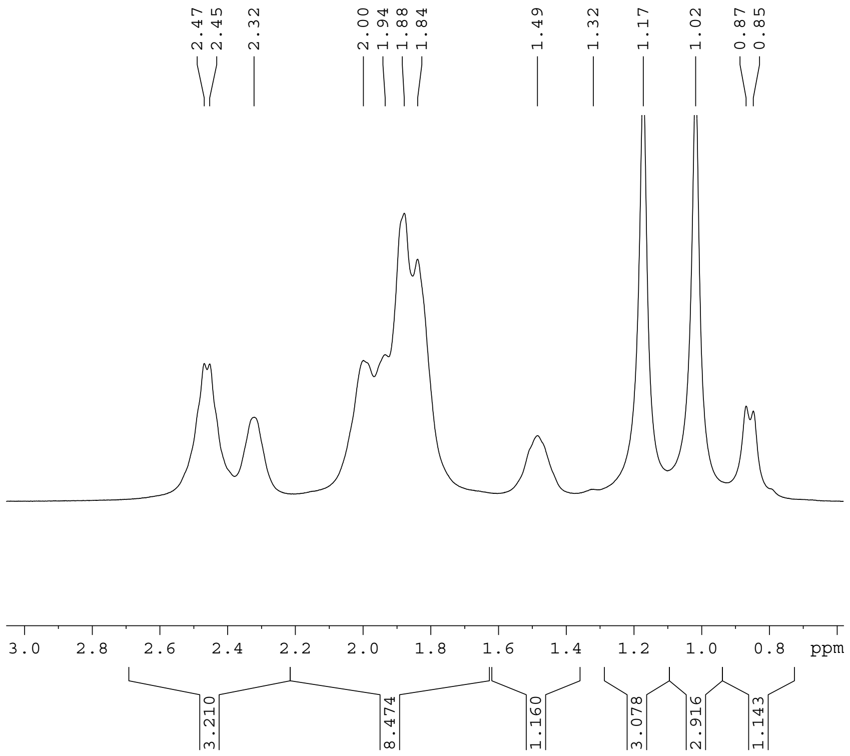

Current Data Parameters
NAME nongda-7
EXPNO 1
PROCNO 1

F2 - Acquisition Parameters
Date_ 20140105
Time 16.41
INSTRUM spect
PROBHD 5 mm QNP 1H/1
PULPROG zg30
TD 65536
SOLVENT CDCl3
NS 4
DS 2
SWH 8278.146 Hz
FIDRES 0.126314 Hz
AQ 3.9584243 sec
RG 50.8
DW 60.400 usec
DE 6.00 usec
TE 288.8 K
D1 2.00000000 sec
MCREST 0.00000000 sec
MCWRK 0.01500000 sec

======== CHANNEL f1 ========
NUC1 1H
P1 14.80 usec
PL1 -2.00 dB
SFO1 400.1332010 MHz

F2 - Processing parameters
SI 32768
SF 400.1300091 MHz
WDW EM
SSB 0
LB 0.30 Hz
GB 0
PC 1.00

^{13}C NMR of compound 8g:

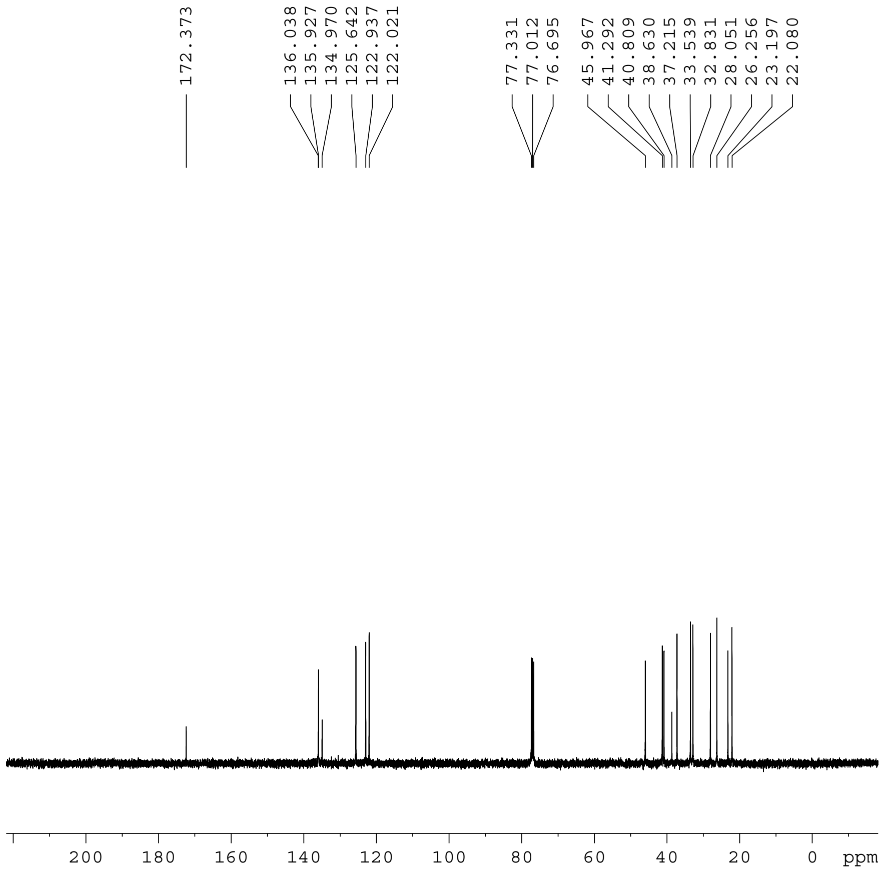

Current Data Parameters
NAME nongda-7
EXPNO 2
PROCNO 1

F2 - Acquisition Parameters
Date_ 20140105
Time 16.44
INSTRUM spect
PROBHD 5 mm QNP 1H/1
PULPROG zgdc30
TD 32768
SOLVENT Acetone
NS 77
DS 0
SWH 24154.590 Hz
FIDRES 0.737140 Hz
AQ 0.6783476 sec
RG 362
DW 20.700 usec
DE 6.00 usec
TE 289.8 K
D1 1.00000000 sec
d11 0.03000000 sec
MCREST 0.00000000 sec
MCWRK 0.01500000 sec

======== CHANNEL f1 ========
NUC1 13C
P1 12.00 usec
PL1 -3.00 dB
SFO1 100.6230315 MHz

======== CHANNEL f2 ========
CPDPRG2 waltz16
NUC2 1H
PCPD2 80.00 usec
PL2 -4.00 dB
PL12 12.12 dB
SFO2 400.1313965 MHz

F2 - Processing parameters
SI 131072
SF 100.6127790 MHz
WDW EM
SSB 0
LB 1.00 Hz
GB 0
PC 0.50

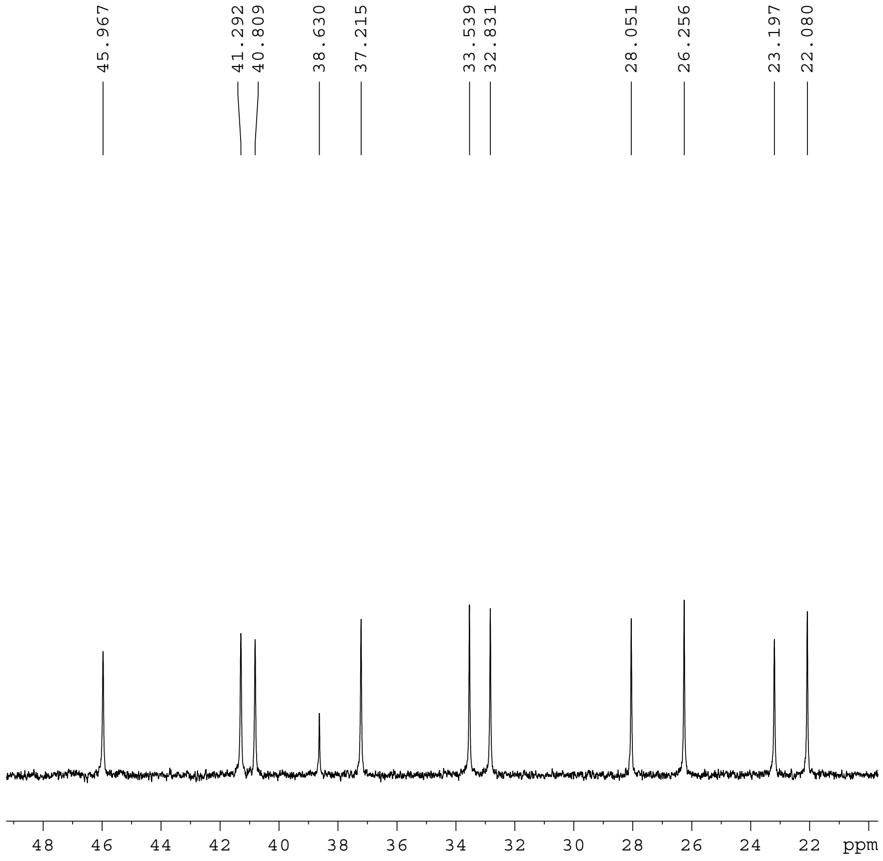

Current Data Parameters
NAME nongda-7
EXPNO 2
PROCNO 1

F2 - Acquisition Parameters
Date_ 20140105
Time 16.44
INSTRUM spect
PROBHD 5 mm QNP 1H/1
PULPROG zgdc30
TD 32768
SOLVENT Acetone
NS 77
DS 0
SWH 24154.590 Hz
FIDRES 0.737140 Hz
AQ 0.6783476 sec
RG 362
DW 20.700 usec
DE 6.00 usec
TE 289.8 K
D1 1.00000000 sec
d11 0.03000000 sec
MCREST 0.00000000 sec
MCWRK 0.01500000 sec

======== CHANNEL f1 ========
NUC1 13C
P1 12.00 usec
PL1 -3.00 dB
SFO1 100.6230315 MHz

======== CHANNEL f2 ========
CPDPRG2 waltz16
NUC2 1H
PCPD2 80.00 usec
PL2 -4.00 dB
PL12 12.12 dB
SFO2 400.1313965 MHz

F2 - Processing parameters
SI 131072
SF 100.6127790 MHz
WDW EM
SSB 0
LB 1.00 Hz
GB 0
PC 0.50

GC-MS spectrums of Compound 8a-8g

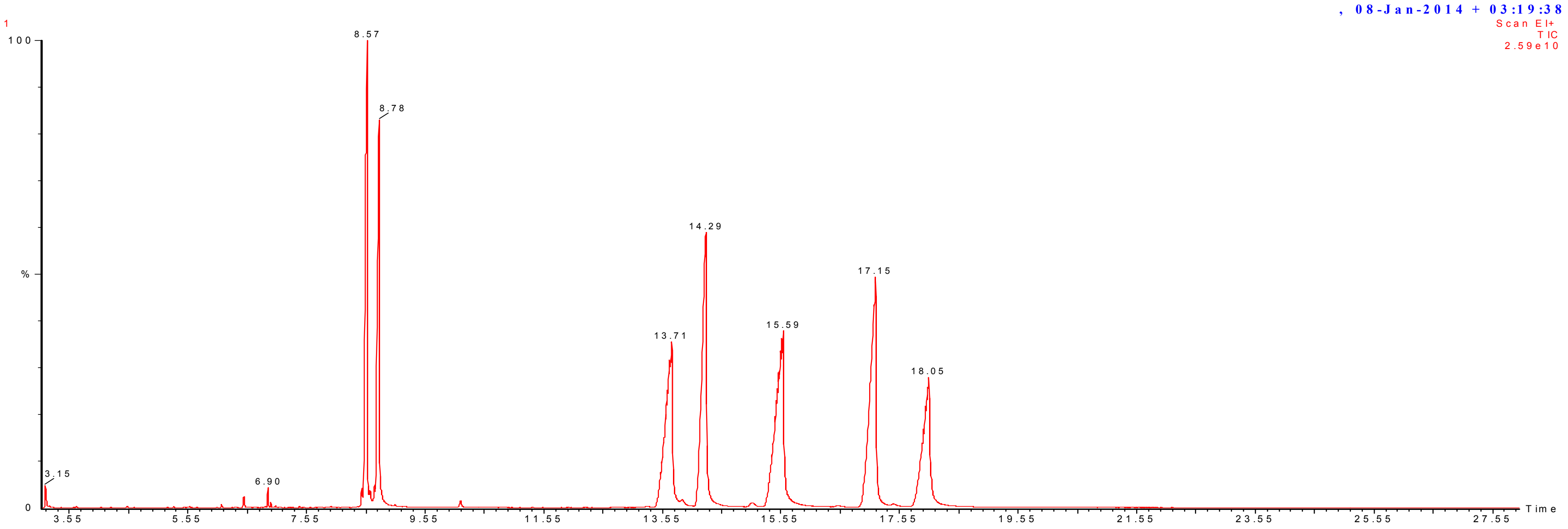

retention time of compounds:

Compound 8a: 8.57 min

Compound 8b: 8.78 min

Compound 8c: 13.71 min

Compound 8d:15.59 min

Compound 8e: 14.29 min

Compound 8f: 18.05 min

Compound 8g: 17.15 min

Compound 8a

1 1094 (8.571) Cm (1082:1097-(1066:1083+1096:1108))

, 08-Jan-2014 + 03:19:38
Scan EI+
1.32e9

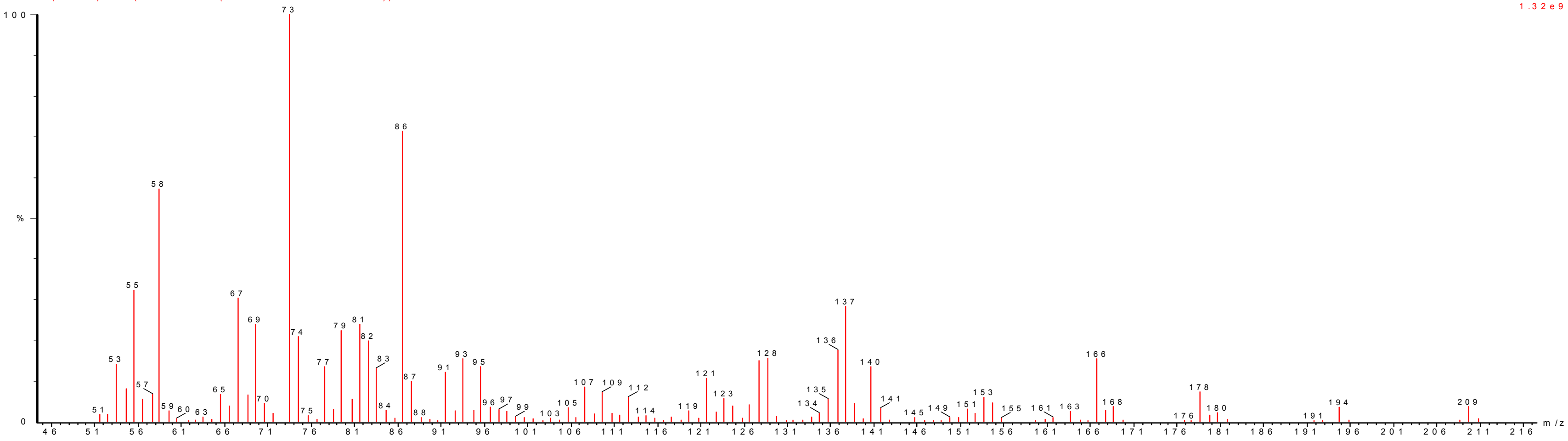

1 1094 (8.571) Cm (1082:1097-(1066:1083+1096:1108))

, 08-Jan-2014 + 03:19:38
Scan EI+

No	Mass	Inten	% BPI	% TIC
1:	51	2.26e7	1.70	0.21
2:	52	2.24e7	1.69	0.21
3:	53	1.85e8	13.96	1.71
4:	54	1.05e8	7.93	0.97
5:	55	4.26e8	32.16	3.93
6:	56	7.18e7	5.43	0.66
7:	57	8.69e7	6.56	0.80
8:	58	7.55e8	57.07	6.98
9:	59	3.41e7	2.58	0.32
10:	60	8.71e6	0.66	0.08
11:	61	2.53e6	0.19	0.02
12:	62	3.49e6	0.26	0.03
13:	63	1.41e7	1.07	0.13
14:	64	5.10e6	0.39	0.05
15:	65	8.84e7	6.68	0.82
16:	66	4.92e7	3.71	0.45
17:	67	4.01e8	30.29	3.71
18:	68	8.61e7	6.50	0.80
19:	69	3.16e8	23.85	2.92
20:	70	5.85e7	4.42	0.54
21:	71	2.60e7	1.97	0.24
22:	73	1.32e9	100.00	12.23
23:	74	2.75e8	20.79	2.54
24:	75	1.80e7	1.36	0.17
25:	76	6.13e6	0.46	0.06
26:	77	1.77e8	13.33	1.63
27:	78	3.74e7	2.82	0.35
28:	79	2.94e8	22.24	2.72
29:	80	7.24e7	5.47	0.67
30:	81	3.14e8	23.76	2.91
31:	82	2.60e8	19.67	2.41
32:	83	1.72e8	13.02	1.59
33:	84	3.60e7	2.72	0.33
34:	85	9.00e6	0.68	0.08
35:	86	9.42e8	71.16	8.70
36:	87	1.30e8	9.83	1.20
37:	88	1.16e7	0.88	0.11
38:	89	6.26e6	0.47	0.06
39:	90	2.87e6	0.22	0.03
40:	91	1.59e8	12.02	1.47
41:	92	3.35e7	2.53	0.31
42:	93	2.04e8	15.43	1.89
43:	94	3.62e7	2.73	0.33
44:	95	1.77e8	13.36	1.63
45:	96	4.63e7	3.50	0.43
46:	97	3.84e7	2.90	0.36
47:	98	3.12e7	2.36	0.29
48:	99	1.42e7	1.07	0.13
49:	100	1.18e7	0.89	0.11
50:	101	8.33e6	0.63	0.08
51:	102	2.07e6	0.16	0.02
52:	103	9.18e6	0.69	0.08
53:	104	4.12e6	0.31	0.04
54:	105	4.40e7	3.32	0.41
55:	106	1.12e7	0.84	0.10
56:	107	1.11e8	8.40	1.03
57:	108	2.41e7	1.82	0.22
58:	109	9.28e7	7.01	0.86
59:	110	2.69e7	2.03	0.25
60:	111	1.93e7	1.46	0.18
61:	112	7.74e7	5.85	0.72
62:	113	1.48e7	1.12	0.14
63:	114	1.78e7	1.35	0.16
64:	115	9.74e6	0.74	0.09
65:	116	2.57e6	0.19	0.02
66:	117	1.36e7	1.03	0.13
67:	118	4.22e6	0.32	0.04
68:	119	3.35e7	2.53	0.31
69:	120	1.01e7	0.76	0.09
70:	121	1.39e8	10.54	1.29
71:	122	3.06e7	2.31	0.28
72:	123	7.31e7	5.52	0.68
73:	124	4.96e7	3.75	0.46
74:	125	1.01e7	0.76	0.09
75:	126	5.47e7	4.13	0.51
76:	127	1.98e8	14.92	1.83
77:	128	2.05e8	15.51	1.90
78:	129	1.51e7	1.14	0.14
79:	130	2.69e6	0.20	0.02
80:	131	4.71e6	0.36	0.04
81:	132	3.19e6	0.24	0.03
82:	133	1.39e7	1.05	0.13
83:	134	2.64e7	2.00	0.24
84:	135	7.24e7	5.47	0.67
85:	136	2.31e8	17.43	2.13
86:	137	3.73e8	28.21	3.45
87:	138	5.79e7	4.38	0.54
88:	139	8.15e6	0.62	0.08
89:	140	1.78e8	13.41	1.64
90:	141	4.09e7	3.09	0.38
91:	142	4.53e6	0.34	0.04
92:	145	1.16e7	0.87	0.11
93:	146	2.61e6	0.20	0.02
94:	147	1.46e6	0.11	0.01
95:	148	1.83e6	0.14	0.02
96:	149	1.12e7	0.85	0.10
97:	150	1.29e7	0.97	0.12
98:	151	3.91e7	2.95	0.36
99:	152	2.56e7	1.93	0.24
100:	153	7.72e7	5.83	0.71
101:	154	5.89e7	4.45	0.54
102:	155	1.07e7	0.81	0.10
103:	159	1.82e6	0.14	0.02
104:	160	6.96e6	0.53	0.06
105:	161	1.12e7	0.85	0.10
106:	163	3.10e7	2.34	0.29
107:	164	4.94e6	0.37	0.05
108:	165	2.38e6	0.18	0.02
109:	166	2.04e8	15.40	1.88
110:	167	3.69e7	2.78	0.34
111:	168	4.87e7	3.68	0.45
112:	169	4.83e6	0.36	0.04
113:	176	2.90e6	0.22	0.03
114:	177	2.33e6	0.18	0.02
115:	178	9.53e7	7.20	0.88
116:	179	1.92e7	1.45	0.18
117:	180	2.74e7	2.07	0.25
118:	181	6.21e6	0.47	0.06
119:	191	2.75e6	0.21	0.03
120:	192	2.57e6	0.19	0.02
121:	194	4.66e7	3.52	0.43
122:	195	4.63e6	0.35	0.04
123:	208	4.40e6	0.33	0.04
124:	209	4.74e7	3.58	0.44
125:	210	7.22e6	0.55	0.07

No Mass Inten % BPI % TIC No Mass Inten % BPI % TIC No Mass Inten % BPI % TIC No Mass

Compound 8b

1 1135 (8.776) Cm (1124:1138-(1108:1125+1138:1155))

, 08-Jan-2014 + 03:19:38
Scan EI+
1.58e9

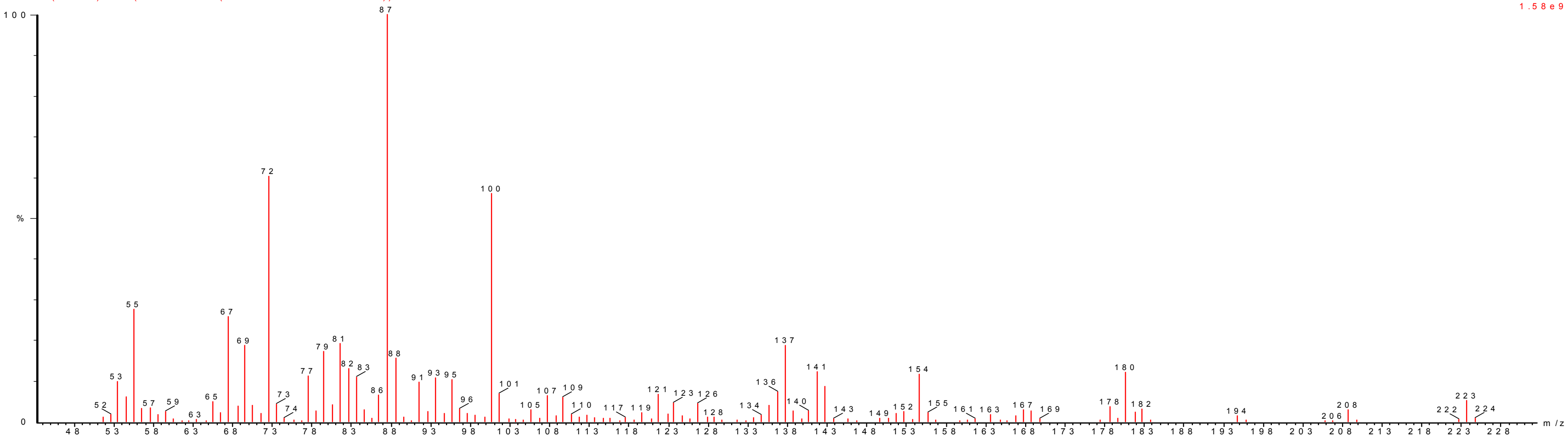

1 1135 (8.776) Cm (1124:1138-(1108:1125+1138:1155))

, 08-Jan-2014 + 03:19:38
Scan EI+

No	Mass	Inten	% BPI	% TIC
1:	51	1.78e7	1.13	0.17
2:	52	2.52e7	1.60	0.24
3:	53	1.54e8	9.74	1.45
4:	54	9.62e7	6.09	0.91
5:	55	4.34e8	27.51	4.09
6:	56	5.03e7	3.19	0.47
7:	57	5.12e7	3.24	0.48
8:	58	2.57e7	1.62	0.24
9:	59	3.88e7	2.46	0.37
10:	60	9.30e6	0.59	0.09
11:	61	2.02e6	0.13	0.02
12:	62	1.81e6	0.11	0.02
13:	63	6.72e6	0.43	0.06
14:	64	3.41e6	0.22	0.03
15:	65	7.59e7	4.81	0.72
16:	66	3.35e7	2.12	0.32
17:	67	4.07e8	25.76	3.83
18:	68	5.89e7	3.73	0.56
19:	69	2.94e8	18.63	2.77
20:	70	6.19e7	3.92	0.58
21:	71	3.16e7	2.00	0.30
22:	72	9.50e8	60.18	8.96
23:	73	6.63e7	4.20	0.63
24:	74	1.19e7	0.75	0.11
25:	75	3.57e6	0.23	0.03
26:	76	3.11e6	0.20	0.03
27:	77	1.75e8	11.11	1.65
28:	78	3.94e7	2.49	0.37
29:	79	2.71e8	17.16	2.55
30:	80	6.51e7	4.12	0.61
31:	81	3.03e8	19.18	2.86
32:	82	2.06e8	13.02	1.94
33:	83	1.71e8	10.86	1.62
34:	84	4.62e7	2.93	0.44
35:	85	1.25e7	0.79	0.12
36:	86	1.02e8	6.45	0.96
37:	87	1.58e9	100.00	14.88
38:	88	2.44e8	15.44	2.30
39:	89	1.70e7	1.08	0.16
40:	90	3.36e6	0.21	0.03
41:	91	1.53e8	9.68	1.44
42:	92	3.92e7	2.48	0.37
43:	93	1.69e8	10.70	1.59
44:	94	3.14e7	1.99	0.30
45:	95	1.63e8	10.31	1.53
46:	96	4.80e7	3.04	0.45
47:	97	3.15e7	1.99	0.30
48:	98	2.33e7	1.48	0.22
49:	99	1.56e7	0.99	0.15
50:	100	8.85e8	56.07	8.34
51:	101	1.08e8	6.84	1.02
52:	102	1.05e7	0.66	0.10
53:	103	7.33e6	0.46	0.07
54:	104	3.88e6	0.25	0.04
55:	105	4.51e7	2.86	0.43
56:	106	1.27e7	0.81	0.12
57:	107	9.88e7	6.26	0.93
58:	108	2.19e7	1.38	0.21
59:	109	9.30e7	5.89	0.88
60:	110	2.52e7	1.60	0.24
61:	111	1.60e7	1.02	0.15
62:	112	2.43e7	1.54	0.23
63:	113	1.33e7	0.84	0.13
64:	114	1.17e7	0.74	0.11
65:	115	1.30e7	0.83	0.12
66:	116	1.95e6	0.12	0.02
67:	117	1.51e7	0.96	0.14
68:	118	4.76e6	0.30	0.04
69:	119	3.28e7	2.08	0.31
70:	120	8.51e6	0.54	0.08
71:	121	1.04e8	6.62	0.98
72:	122	2.78e7	1.76	0.26
73:	123	7.04e7	4.46	0.66
74:	124	2.24e7	1.42	0.21
75:	125	9.01e6	0.57	0.08
76:	126	6.85e7	4.34	0.65
77:	127	1.61e7	1.02	0.15
78:	128	1.62e7	1.03	0.15
79:	129	4.91e6	0.31	0.05
80:	131	3.74e6	0.24	0.04
81:	132	2.04e6	0.13	0.02
82:	133	1.38e7	0.87	0.13
83:	134	2.36e7	1.49	0.22
84:	135	6.08e7	3.85	0.57
85:	136	1.15e8	7.26	1.08
86:	137	2.95e8	18.72	2.79
87:	138	4.10e7	2.60	0.39
88:	139	8.53e6	0.54	0.08
89:	140	4.11e7	2.60	0.39
90:	141	1.93e8	12.25	1.82
91:	142	1.36e8	8.59	1.28
92:	143	1.07e7	0.68	0.10
93:	145	9.34e6	0.59	0.09
94:	146	1.93e6	0.12	0.02
95:	149	1.29e7	0.81	0.12
96:	150	1.19e7	0.76	0.11
97:	151	3.02e7	1.91	0.28
98:	152	3.85e7	2.44	0.36
99:	153	7.99e6	0.51	0.08
100:	154	1.82e8	11.54	1.72
101:	155	3.40e7	2.15	0.32
102:	156	4.17e6	0.26	0.04
103:	159	2.55e6	0.16	0.02
104:	160	4.28e6	0.27	0.04
105:	161	1.01e7	0.64	0.10
106:	163	2.71e7	1.72	0.26
107:	164	3.94e6	0.25	0.04
108:	165	2.42e6	0.15	0.02
109:	166	2.11e7	1.34	0.20
110:	167	4.53e7	2.87	0.43
111:	168	3.97e7	2.51	0.37
112:	169	8.98e6	0.57	0.08
113:	177	4.56e6	0.29	0.04
114:	178	5.79e7	3.67	0.55
115:	179	1.14e7	0.72	0.11
116:	180	1.89e8	12.00	1.79
117:	181	3.56e7	2.26	0.34
118:	182	4.77e7	3.02	0.45
119:	183	5.04e6	0.32	0.05
120:	194	2.23e7	1.41	0.21
121:	195	5.42e6	0.34	0.05
122:	205	1.61e6	0.10	0.02
123:	206	2.52e6	0.16	0.02
124:	208	4.55e7	2.88	0.43
125:	209	5.57e6	0.35	0.05
126:	222	7.34e6	0.47	0.07
127:	223	8.07e7	5.11	0.76
128:	224	1.20e7	0.76	0.11

Compound 8c

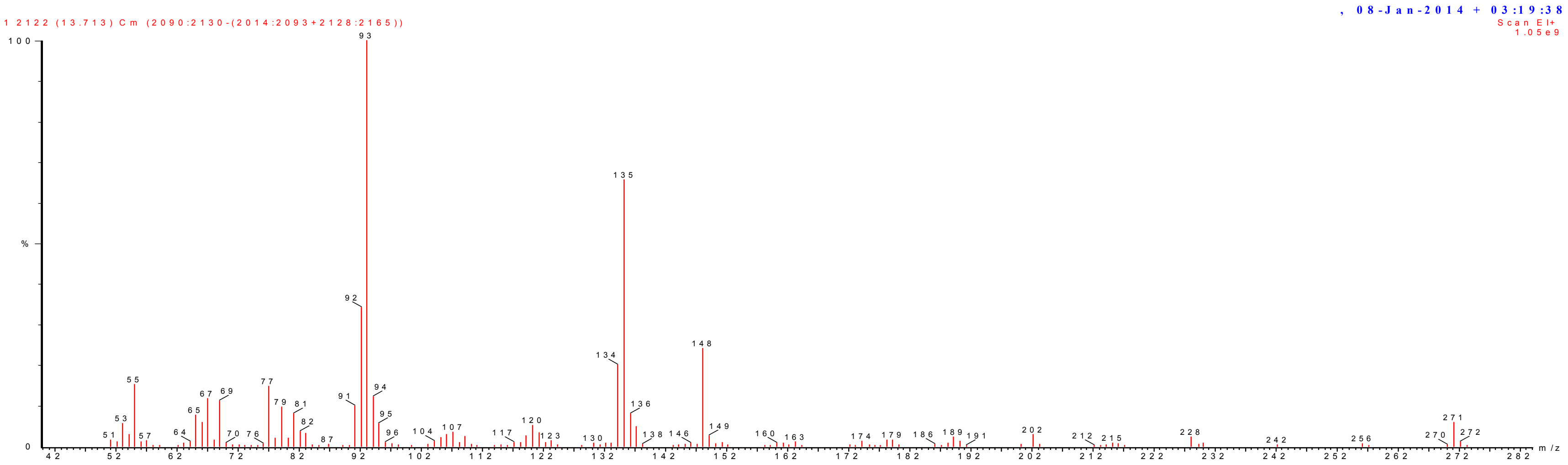

1 2122 (13.713) Cm (2090:2130-(2014:2093+2128:2165))

, 08-Jan-2014 + 03:19:38
Scan EI+

No	Mass	Inten	% BPI	% TIC
1:	51	1.63e7	1.55	0.33
2:	52	1.04e7	0.99	0.21
3:	53	5.79e7	5.50	1.16
4:	54	3.02e7	2.86	0.60
5:	55	1.60e8	15.20	3.20
6:	56	1.08e7	1.02	0.22
7:	57	1.39e7	1.32	0.28
8:	58	1.33e6	0.13	0.03
9:	59	1.18e6	0.11	0.02
10:	62	1.50e6	0.14	0.03
11:	63	8.09e6	0.77	0.16
12:	64	1.13e7	1.07	0.23
13:	65	8.16e7	7.75	1.63
14:	66	6.21e7	5.90	1.24
15:	67	1.23e8	11.68	2.46
16:	68	1.59e7	1.51	0.32
17:	69	1.17e8	11.07	2.33
18:	70	7.67e6	0.73	0.15
19:	71	2.96e6	0.28	0.06
20:	72	2.48e6	0.24	0.05
21:	73	2.22e6	0.21	0.04
22:	74	1.11e6	0.11	0.02
23:	75	1.50e6	0.14	0.03
24:	76	6.08e6	0.58	0.12
25:	77	1.56e8	14.81	3.12
26:	78	2.07e7	1.97	0.41
27:	79	1.02e8	9.71	2.04
28:	80	1.99e7	1.89	0.40
29:	81	8.41e7	7.99	1.68
30:	82	3.87e7	3.67	0.77
31:	83	3.26e7	3.10	0.65
32:	84	3.54e6	0.34	0.07
33:	85	1.63e6	0.15	0.03
34:	87	5.03e6	0.48	0.10
35:	89	1.83e6	0.17	0.04
36:	90	2.23e6	0.21	0.04
37:	91	1.05e8	9.95	2.09
38:	92	3.60e8	34.17	7.19
39:	93	1.05e9	100.00	21.05
40:	94	1.29e8	12.26	2.58
41:	95	5.83e7	5.53	1.17
42:	96	9.75e6	0.93	0.19
43:	97	6.51e6	0.62	0.13
44:	98	2.39e6	0.23	0.05
45:	100	1.87e6	0.18	0.04
46:	103	4.31e6	0.41	0.09
47:	104	1.23e7	1.17	0.25
48:	105	2.18e7	2.07	0.44
49:	106	2.99e7	2.84	0.60
50:	107	3.68e7	3.49	0.73
51:	108	8.92e6	0.85	0.18
52:	109	2.51e7	2.38	0.50
53:	110	5.10e6	0.48	0.10
54:	111	2.34e6	0.22	0.05
55:	114	1.36e6	0.13	0.03
56:	115	3.92e6	0.37	0.08
57:	116	1.70e6	0.16	0.03
58:	117	1.03e7	0.98	0.21
59:	118	9.29e6	0.88	0.19
60:	119	2.63e7	2.50	0.53
61:	120	5.36e7	5.09	1.07
62:	121	3.54e7	3.36	0.71
63:	122	9.24e6	0.88	0.18
64:	123	1.39e7	1.32	0.28
65:	124	3.76e6	0.36	0.08
66:	128	2.21e6	0.21	0.04
67:	130	8.54e6	0.81	0.17
68:	131	3.59e6	0.34	0.07
69:	132	8.52e6	0.81	0.17
70:	133	8.37e6	0.79	0.17
71:	134	2.11e8	20.03	4.22
72:	135	6.92e8	65.72	13.83
73:	136	8.39e7	7.96	1.68
74:	137	5.05e7	4.80	1.01
75:	138	5.18e6	0.49	0.10
76:	143	1.26e6	0.12	0.03
77:	144	2.61e6	0.25	0.05
78:	145	4.22e6	0.40	0.08
79:	146	7.65e6	0.73	0.15
80:	147	4.46e6	0.42	0.09
81:	148	2.54e8	24.08	5.07
82:	149	2.65e7	2.52	0.53
83:	150	5.79e6	0.55	0.12
84:	151	1.01e7	0.96	0.20
85:	152	3.49e6	0.33	0.07
86:	158	2.13e6	0.20	0.04
87:	159	1.85e6	0.18	0.04
88:	160	8.34e6	0.79	0.17
89:	161	7.32e6	0.70	0.15
90:	162	2.93e6	0.28	0.06
91:	163	1.19e7	1.13	0.24
92:	164	1.26e6	0.12	0.03
93:	172	2.71e6	0.26	0.05
94:	173	1.06e6	0.10	0.02
95:	174	1.22e7	1.16	0.24
96:	175	2.41e6	0.23	0.05
97:	176	1.58e6	0.15	0.03
98:	177	1.63e6	0.15	0.03
99:	178	1.55e7	1.47	0.31
100:	179	1.66e7	1.58	0.33
101:	180	3.28e6	0.31	0.07
102:	186	4.52e6	0.43	0.09
103:	187	2.01e6	0.19	0.04
104:	188	7.41e6	0.70	0.15
105:	189	2.37e7	2.25	0.47
106:	190	1.34e7	1.27	0.27
107:	191	1.46e6	0.14	0.03
108:	200	4.29e6	0.41	0.09
109:	202	2.98e7	2.83	0.60
110:	203	4.36e6	0.41	0.09
111:	212	1.89e6	0.18	0.04
112:	213	1.82e6	0.17	0.04
113:	214	3.33e6	0.32	0.07
114:	215	7.36e6	0.70	0.15
115:	216	6.82e6	0.65	0.14
116:	217	1.46e6	0.14	0.03
117:	228	2.34e7	2.22	0.47
118:	229	5.05e6	0.48	0.10
119:	230	8.60e6	0.82	0.17
120:	242	2.85e6	0.27	0.06
121:	256	6.58e6	0.63	0.13
122:	257	1.06e6	0.10	0.02
123:	270	2.74e6	0.26	0.05
124:	271	6.23e7	5.92	1.25
125:	272	1.10e7	1.05	0.22
126:	273	1.13e6	0.11	0.02

Compound 8d

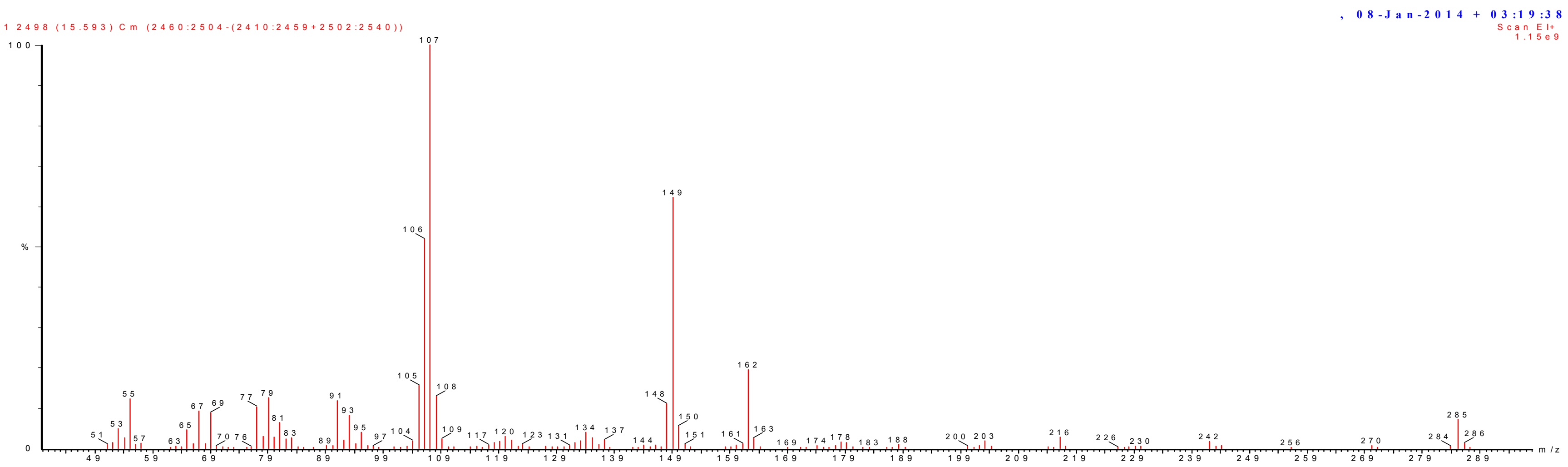

1 2498 (15.593) Cm (2460:2504-(2410:2459+2502:2540))

, 08-Jan-2014 + 03:19:38

Scan EI+

No	Mass	Inten	% BPI	% TIC
1:	51	1.07e7	0.93	0.20
2:	52	1.48e7	1.28	0.27
3:	53	5.62e7	4.88	1.04
4:	54	2.93e7	2.55	0.54
5:	55	1.40e8	12.16	2.60
6:	56	9.57e6	0.83	0.18
7:	57	1.34e7	1.17	0.25
8:	62	1.29e6	0.11	0.02
9:	63	5.81e6	0.50	0.11
10:	64	3.93e6	0.34	0.07
11:	65	5.22e7	4.54	0.97
12:	66	1.30e7	1.13	0.24
13:	67	1.06e8	9.19	1.96
14:	68	1.25e7	1.09	0.23
15:	69	1.00e8	8.70	1.86
16:	70	5.69e6	0.49	0.11
17:	71	2.69e6	0.23	0.05
18:	72	1.83e6	0.16	0.03
19:	73	2.23e6	0.19	0.04
20:	75	1.51e6	0.13	0.03
21:	76	3.05e6	0.27	0.06
22:	77	1.17e8	10.19	2.18
23:	78	3.37e7	2.93	0.63
24:	79	1.44e8	12.51	2.67
25:	80	3.13e7	2.71	0.58
26:	81	7.32e7	6.36	1.36
27:	82	2.58e7	2.24	0.48
28:	83	2.96e7	2.57	0.55
29:	84	2.79e6	0.24	0.05
30:	85	1.51e6	0.13	0.03
31:	87	2.35e6	0.20	0.04
32:	89	7.04e6	0.61	0.13
33:	90	6.50e6	0.56	0.12
34:	91	1.35e8	11.74	2.51
35:	92	2.34e7	2.03	0.43
36:	93	9.31e7	8.08	1.73
37:	94	1.28e7	1.12	0.24
38:	95	4.45e7	3.87	0.83
39:	96	6.93e6	0.60	0.13
40:	97	5.24e6	0.45	0.10
41:	98	2.23e6	0.19	0.04
42:	101	3.62e6	0.31	0.07
43:	102	1.44e6	0.13	0.03
44:	103	6.03e6	0.52	0.11
45:	104	2.03e7	1.76	0.38
46:	105	1.79e8	15.50	3.31
47:	106	5.96e8	51.80	11.06
48:	107	1.15e9	100.00	21.35
49:	108	1.47e8	12.73	2.72
50:	109	2.59e7	2.25	0.48
51:	110	4.16e6	0.36	0.08
52:	111	2.67e6	0.23	0.05
53:	114	3.04e6	0.26	0.06
54:	115	5.87e6	0.51	0.11
55:	116	1.61e6	0.14	0.03
56:	117	7.98e6	0.69	0.15
57:	118	1.49e7	1.29	0.28
58:	119	1.89e7	1.64	0.35
59:	120	3.37e7	2.93	0.63
60:	121	2.27e7	1.98	0.42
61:	122	5.82e6	0.51	0.11
62:	123	1.09e7	0.95	0.20
63:	124	2.73e6	0.24	0.05
64:	127	5.96e6	0.52	0.11
65:	128	2.72e6	0.24	0.05
66:	129	3.67e6	0.32	0.07
67:	130	4.16e6	0.36	0.08
68:	131	6.37e6	0.55	0.12
69:	132	1.51e7	1.31	0.28
70:	133	2.14e7	1.85	0.40
71:	134	4.45e7	3.87	0.83
72:	135	2.97e7	2.58	0.55
73:	136	1.08e7	0.94	0.20
74:	137	2.30e7	2.00	0.43
75:	138	2.54e6	0.22	0.05
76:	142	1.49e6	0.13	0.03
77:	143	1.17e6	0.10	0.02
78:	144	8.02e6	0.70	0.15
79:	145	3.87e6	0.34	0.07
80:	146	7.85e6	0.68	0.15
81:	147	3.09e6	0.27	0.06
82:	148	1.26e8	10.95	2.34
83:	149	7.15e8	62.13	13.27
84:	150	6.23e7	5.41	1.16
85:	151	9.81e6	0.85	0.18
86:	152	3.72e6	0.32	0.07
87:	158	2.63e6	0.23	0.05
88:	159	3.13e6	0.27	0.06
89:	160	7.88e6	0.68	0.15
90:	161	1.16e7	1.01	0.21
91:	162	2.24e8	19.41	4.14
92:	163	2.73e7	2.37	0.51
93:	164	2.66e6	0.23	0.05
94:	169	2.33e6	0.20	0.04
95:	171	1.21e6	0.11	0.02
96:	172	2.08e6	0.18	0.04
97:	174	6.75e6	0.59	0.13
98:	175	2.24e6	0.19	0.04
99:	176	1.85e6	0.16	0.03
100:	177	7.37e6	0.64	0.14
101:	178	1.69e7	1.47	0.31
102:	179	1.50e7	1.30	0.28
103:	180	3.63e6	0.32	0.07
104:	182	1.57e6	0.14	0.03
105:	183	1.60e6	0.14	0.03
106:	186	2.53e6	0.22	0.05
107:	187	1.20e6	0.10	0.02
108:	188	1.08e7	0.94	0.20
109:	189	2.35e6	0.20	0.04
110:	200	4.90e6	0.43	0.09
111:	201	1.93e6	0.17	0.04
112:	202	6.14e6	0.53	0.11
113:	203	2.07e7	1.80	0.38
114:	204	4.76e6	0.41	0.09
115:	214	3.98e6	0.35	0.07
116:	215	1.21e6	0.10	0.02
117:	216	3.19e7	2.77	0.59
118:	217	4.93e6	0.43	0.09
119:	226	2.31e6	0.20	0.04
120:	227	2.30e6	0.20	0.04
121:	228	2.64e6	0.23	0.05
122:	229	4.61e6	0.40	0.09
123:	230	4.66e6	0.40	0.09
124:	242	1.99e7	1.73	0.37
125:	243	4.66e6	0.41	0.09
126:	244	6.77e6	0.59	0.13
127:	256	2.43e6	0.21	0.05
128:	270	6.69e6	0.58	0.12
129:	271	1.37e6	0.12	0.03
130:	284	4.83e6	0.42	0.09
131:	285	8.07e7	7.01	1.50
132:	286	1.37e7	1.19	0.25
133:	287	1.42e6	0.12	0.03

Compound 8e

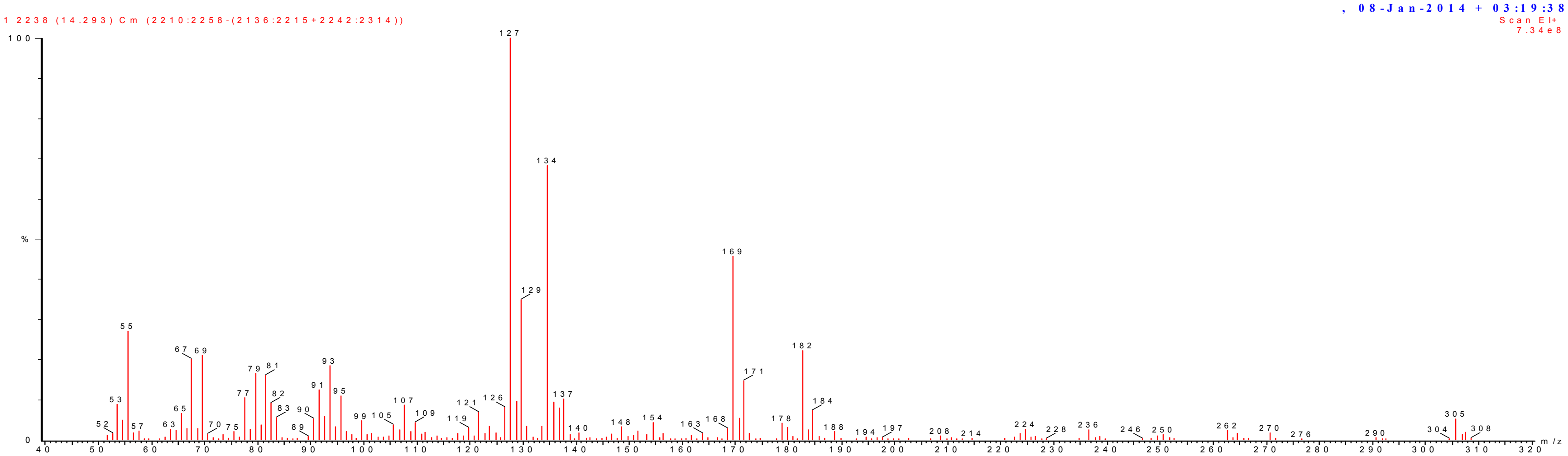

1 2238 (14.293) Cm (2210:2258-(2136:2215+2242:2314))

, 08-Jan-2014 + 03:19:38

No	Mass	Inten	% BPI	% TIC
1:	51	7.93e6	1.08	0.15
2:	52	1.12e7	1.52	0.21
3:	53	6.41e7	8.73	1.21
4:	54	3.50e7	4.77	0.66
5:	55	1.98e8	26.93	3.72
6:	56	1.27e7	1.73	0.24
7:	57	1.50e7	2.04	0.28
8:	58	1.31e6	0.18	0.02
9:	59	1.18e6	0.16	0.02
10:	61	1.25e6	0.17	0.02
11:	62	3.96e6	0.54	0.07
12:	63	1.92e7	2.62	0.36
13:	64	1.67e7	2.27	0.31
14:	65	4.79e7	6.52	0.90
15:	66	1.94e7	2.64	0.37
16:	67	1.48e8	20.10	2.78
17:	68	2.00e7	2.72	0.38
18:	69	1.54e8	20.93	2.89
19:	70	9.48e6	1.29	0.18
20:	71	3.52e6	0.48	0.07
21:	72	1.37e6	0.19	0.03
22:	73	8.76e6	1.19	0.16
23:	74	2.50e6	0.34	0.05
24:	75	1.44e7	1.96	0.27
25:	76	4.00e6	0.55	0.08
26:	77	7.68e7	10.47	1.45
27:	78	1.88e7	2.56	0.35
28:	79	1.21e8	16.49	2.28
29:	80	2.64e7	3.59	0.50
30:	81	1.17e8	15.96	2.20
31:	82	6.68e7	9.10	1.26
32:	83	4.03e7	5.49	0.76
33:	84	3.79e6	0.52	0.07
34:	85	2.08e6	0.28	0.04
35:	86	8.69e5	0.12	0.02
36:	87	1.89e6	0.26	0.04
37:	89	6.02e6	0.82	0.11
38:	90	3.81e7	5.20	0.72
39:	91	9.11e7	12.41	1.71
40:	92	4.18e7	5.69	0.79
41:	93	1.35e8	18.36	2.54
42:	94	2.27e7	3.10	0.43
43:	95	7.95e7	10.83	1.50
44:	96	1.42e7	1.93	0.27
45:	97	9.23e6	1.26	0.17
46:	98	2.53e6	0.34	0.05
47:	99	3.47e7	4.73	0.65
48:	100	9.02e6	1.23	0.17
49:	101	1.15e7	1.57	0.22
50:	102	3.92e6	0.53	0.07
51:	103	4.84e6	0.66	0.09
52:	104	6.11e6	0.83	0.11
53:	105	2.60e7	3.54	0.49
54:	106	1.71e7	2.33	0.32
55:	107	6.28e7	8.56	1.18
56:	108	1.46e7	1.99	0.27
57:	109	3.12e7	4.25	0.59
58:	110	9.81e6	1.34	0.18
59:	111	1.37e7	1.86	0.26
60:	112	2.83e6	0.39	0.05
61:	113	6.55e6	0.89	0.12
62:	114	2.02e6	0.28	0.04
63:	115	3.19e6	0.43	0.06
64:	116	2.60e6	0.35	0.05
65:	117	1.05e7	1.44	0.20
66:	118	7.04e6	0.96	0.13
67:	119	2.11e7	2.87	0.40
68:	120	6.43e6	0.88	0.12
69:	121	5.00e7	6.81	0.94
70:	122	1.13e7	1.53	0.21
71:	123	2.45e7	3.34	0.46
72:	124	1.16e7	1.58	0.22
73:	125	3.83e6	0.52	0.07
74:	126	5.88e7	8.00	1.11
75:	127	7.34e8	100.00	13.81
76:	128	7.01e7	9.55	1.32
77:	129	2.55e8	34.76	4.80
78:	130	2.39e7	3.26	0.45
79:	131	4.86e6	0.66	0.09
80:	132	1.89e6	0.26	0.04
81:	133	2.39e7	3.26	0.45
82:	134	5.00e8	68.19	9.42
83:	135	6.89e7	9.38	1.30
84:	136	5.73e7	7.81	1.08
85:	137	7.38e7	10.06	1.39
86:	138	9.13e6	1.24	0.17
87:	139	9.38e5	0.13	0.02
88:	140	1.19e7	1.62	0.22
89:	141	2.06e6	0.28	0.04
90:	142	3.27e6	0.45	0.06
91:	143	1.36e6	0.19	0.03
92:	144	2.44e6	0.33	0.05
93:	145	4.60e6	0.63	0.09
94:	146	9.88e6	1.35	0.19
95:	147	2.65e6	0.36	0.05
96:	148	2.30e7	3.14	0.43
97:	149	5.94e6	0.81	0.11
98:	150	7.36e6	1.00	0.14
99:	151	1.59e7	2.17	0.30
100:	153	8.58e6	1.17	0.16
101:	154	3.14e7	4.28	0.59
102:	155	2.83e6	0.39	0.05
103:	156	1.14e7	1.56	0.21
104:	157	1.15e6	0.16	0.02
105:	158	1.37e6	0.19	0.03
106:	159	1.36e6	0.19	0.03
107:	160	2.49e6	0.34	0.05
108:	161	7.94e6	1.08	0.15
109:	162	1.31e6	0.18	0.02
110:	163	1.16e7	1.58	0.22
111:	164	3.34e6	0.45	0.06
112:	166	3.31e6	0.45	0.06
113:	167	9.04e5	0.12	0.02
114:	168	2.02e7	2.76	0.38
115:	169	3.35e8	45.60	6.30
116:	170	3.83e7	5.22	0.72
117:	171	1.08e8	14.68	2.03
118:	172	1.08e7	1.47	0.20
119:	173	1.19e6	0.16	0.02
120:	174	2.40e6	0.33	0.05
121:	177	1.48e6	0.20	0.03
122:	178	2.96e7	4.03	0.56
123:	179	2.23e7	3.04	0.42
124:	180	5.56e6	0.76	0.10
125:	181	1.35e6	0.18	0.03
126:	182	1.62e8	22.09	3.05
127:	183	1.79e7	2.44	0.34
128:	184	5.31e7	7.23	1.00
129:	185	5.14e6	0.70	0.10
130:	186	1.78e6	0.24	0.03
131:	188	1.48e7	2.02	0.28
132:	189	1.86e6	0.25	0.04
133:	192	9.61e5	0.13	0.02
134:	194	4.76e6	0.65	0.09
135:	195	1.15e6	0.16	0.02
136:	196	3.31e6	0.45	0.06
137:	197	3.13e6	0.43	0.06
138:	198	1.15e6	0.16	0.02
139:	199	1.04e6	0.14	0.02
140:	200	1.49e6	0.20	0.03
141:	202	1.85e6	0.25	0.03
142:	206	1.48e6	0.20	0.03
143:	208	7.09e6	0.97	0.13
144:	209	1.34e6	0.18	0.03
145:	210	3.46e6	0.47	0.07
146:	211	8.70e5	0.12	0.02
147:	212	1.27e6	0.17	0.02
148:	214	2.28e6	0.31	0.04
149:	220	2.27e6	0.31	0.04
150:	222	4.83e6	0.66	0.09
151:	223	1.10e7	1.50	0.21
152:	224	1.89e7	2.58	0.36
153:	225	4.83e6	0.66	0.09
154:	226	5.51e6	0.75	0.10
155:	227	8.01e5	0.11	0.02
156:	228	8.44e5	0.11	0.02
157:	234	2.75e6	0.37	0.05
158:	236	1.82e7	2.48	0.34
159:	237	2.83e6	0.39	0.05
160:	238	5.88e6	0.80	0.11
161:	239	8.14e5	0.11	0.02
162:	246	1.03e6	0.14	0.02
163:	248	2.73e6	0.37	0.05
164:	249	6.72e6	0.92	0.13
165:	250	8.73e6	1.19	0.16
166:	251	3.21e6	0.44	0.06
167:	252	2.54e6	0.35	0.05
168:	262	1.62e7	2.21	0.30
169:	263	3.04e6	0.41	0.06
170:	264	1.08e7	1.47	0.20
171:	265	1.89e6	0.26	0.04
172:	266	1.83e6	0.25	0.03
173:	270	1.18e7	1.61	0.22
174:	271	1.98e6	0.27	0.04
175:	276	1.64e6	0.22	0.03
176:	290	3.63e6	0.49	0.07
177:	291	7.74e5	0.11	0.01
178:	292	1.20e6	0.16	0.02
179:	304	8.51e5	0.12	0.02
180:	305	3.77e7	5.13	0.71
181:	306	9.07e6	1.24	0.17
182:	307	1.29e7	1.75	0.24
183:	308	2.13e6	0.29	0.04

Compound 8f

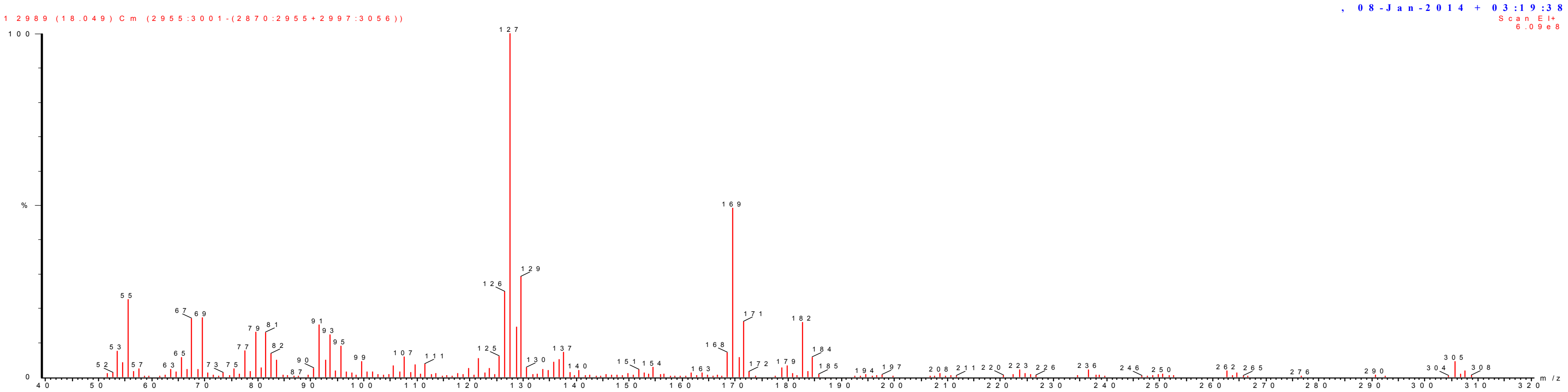

1 2989 (18.049) Cm (2955:3001-(2870:2955+2997:3056))

, 08-Jan-2014 + 03:19:38
Scan EI+

No	Mass	Inten	% BPI	% TIC
1:	51	5.71e6	0.94	0.16
2:	52	6.00e6	0.99	0.17
3:	53	4.50e7	7.40	1.25
4:	54	2.44e7	4.00	0.68
5:	55	1.36e8	22.42	3.79
6:	56	9.34e6	1.53	0.26
7:	57	1.37e7	2.25	0.38
8:	58	1.13e6	0.19	0.03
9:	59	1.18e6	0.19	0.03
10:	61	9.04e5	0.15	0.03
11:	62	3.09e6	0.51	0.09
12:	63	1.28e7	2.11	0.36
13:	64	8.42e6	1.38	0.23
14:	65	3.40e7	5.59	0.95
15:	66	1.29e7	2.12	0.36
16:	67	1.03e8	16.88	2.86
17:	68	1.30e7	2.14	0.36
18:	69	1.05e8	17.22	2.91
19:	70	6.39e6	1.05	0.18
20:	71	2.67e6	0.44	0.07
21:	72	1.36e6	0.22	0.04
22:	73	6.77e6	1.11	0.19
23:	74	1.87e6	0.31	0.05
24:	75	1.36e7	2.23	0.38
25:	76	4.65e6	0.76	0.13
26:	77	4.59e7	7.55	1.28
27:	78	9.55e6	1.57	0.27
28:	79	7.91e7	13.00	2.20
29:	80	1.57e7	2.57	0.44
30:	81	7.78e7	12.78	2.16
31:	82	4.03e7	6.62	1.12
32:	83	2.89e7	4.75	0.80
33:	84	2.91e6	0.48	0.08
34:	85	2.04e6	0.33	0.06
35:	86	6.68e5	0.11	0.02
36:	87	7.77e5	0.13	0.02
37:	89	3.06e6	0.50	0.09
38:	90	1.47e7	2.42	0.41
39:	91	9.14e7	15.02	2.54
40:	92	2.90e7	4.76	0.81
41:	93	7.44e7	12.22	2.07
42:	94	9.80e6	1.61	0.27
43:	95	5.41e7	8.89	1.51
44:	96	8.41e6	1.38	0.23
45:	97	6.84e6	1.12	0.19
46:	98	2.48e6	0.41	0.07
47:	99	2.62e7	4.30	0.73
48:	100	8.42e6	1.38	0.23
49:	101	7.99e6	1.31	0.22
50:	102	3.44e6	0.57	0.10
51:	103	3.11e6	0.51	0.09
52:	104	3.85e6	0.63	0.11
53:	105	1.91e7	3.14	0.53
54:	106	8.43e6	1.38	0.23
55:	107	3.51e7	5.77	0.98
56:	108	7.08e6	1.16	0.20
57:	109	2.13e7	3.50	0.59
58:	110	4.65e6	0.76	0.13
59:	111	2.16e7	3.54	0.60
60:	112	3.30e6	0.54	0.09
61:	113	5.69e6	0.94	0.16
62:	114	9.25e5	0.15	0.03
63:	115	2.03e6	0.33	0.06
64:	116	1.35e6	0.22	0.04
65:	117	5.87e6	0.96	0.16
66:	118	3.25e6	0.53	0.09
67:	119	1.46e7	2.39	0.40
68:	120	3.00e6	0.49	0.08
69:	121	3.24e7	5.32	0.90
70:	122	6.68e6	1.10	0.19
71:	123	1.51e7	2.47	0.42
72:	124	3.74e6	0.61	0.10
73:	125	3.54e7	5.81	0.98
74:	126	1.50e8	24.65	4.17
75:	127	6.09e8	100.00	16.92
76:	128	8.79e7	14.45	2.44
77:	129	1.77e8	29.09	4.92
78:	130	1.52e7	2.49	0.42
79:	131	3.64e6	0.60	0.10
80:	132	4.15e6	0.68	0.12
81:	133	1.27e7	2.08	0.35
82:	134	1.07e7	1.76	0.30
83:	135	2.57e7	4.21	0.71
84:	136	3.07e7	5.04	0.85
85:	137	4.27e7	7.02	1.19
86:	138	7.34e6	1.21	0.20
87:	139	2.08e6	0.34	0.06
88:	140	1.09e7	1.79	0.30
89:	141	1.79e6	0.29	0.05
90:	142	3.14e6	0.52	0.09
91:	143	1.12e6	0.18	0.03
92:	144	1.27e6	0.21	0.04
93:	145	3.65e6	0.60	0.10
94:	146	2.34e6	0.39	0.07
95:	147	2.65e6	0.44	0.07
96:	148	1.52e6	0.25	0.04
97:	149	5.44e6	0.89	0.15
98:	150	3.18e6	0.52	0.09
99:	151	1.22e7	2.01	0.34
100:	152	6.06e6	1.00	0.17
101:	153	4.27e6	0.70	0.12
102:	154	1.65e7	2.72	0.46
103:	155	3.24e6	0.53	0.09
104:	156	4.83e6	0.79	0.13
105:	157	8.07e5	0.13	0.02
106:	158	6.58e5	0.11	0.02
107:	159	1.01e6	0.17	0.03
108:	160	6.70e5	0.11	0.02
109:	161	6.69e6	1.10	0.19
110:	162	1.70e6	0.28	0.05
111:	163	6.70e6	1.10	0.19
112:	164	2.87e6	0.47	0.08
113:	165	8.42e5	0.14	0.02
114:	166	2.98e6	0.49	0.08
115:	167	1.03e6	0.17	0.03
116:	168	4.25e7	6.99	1.18
117:	169	2.99e8	49.13	8.31
118:	170	3.39e7	5.57	0.94
119:	171	9.76e7	16.04	2.71
120:	172	8.18e6	1.34	0.23
121:	173	7.99e5	0.13	0.02
122:	177	9.65e5	0.16	0.03
123:	178	1.52e7	2.50	0.42
124:	179	1.96e7	3.22	0.54
125:	180	5.62e6	0.92	0.16
126:	181	1.72e6	0.28	0.05
127:	182	9.66e7	15.88	2.69
128:	183	9.10e6	1.49	0.25
129:	184	3.39e7	5.57	0.94
130:	185	3.41e6	0.56	0.09
131:	192	9.11e5	0.15	0.03
132:	193	6.33e5	0.10	0.02
133:	194	3.72e6	0.61	0.10
134:	195	1.06e6	0.17	0.03
135:	196	2.04e6	0.34	0.06
136:	197	2.48e6	0.41	0.07
137:	199	1.04e6	0.17	0.03
138:	206	1.07e6	0.18	0.03
139:	207	6.90e5	0.11	0.02
140:	208	5.33e6	0.88	0.15
141:	209	1.14e6	0.19	0.03
142:	210	2.08e6	0.34	0.06
143:	211	6.25e5	0.10	0.02
144:	220	2.04e6	0.34	0.06
145:	222	3.96e6	0.65	0.11
146:	223	1.16e7	1.90	0.32
147:	224	5.20e6	0.85	0.14
148:	225	3.85e6	0.63	0.11
149:	226	1.49e6	0.25	0.04
150:	234	1.83e6	0.30	0.05
151:	236	1.18e7	1.93	0.33
152:	237	2.70e6	0.44	0.08
153:	238	3.13e6	0.51	0.09
154:	239	6.36e5	0.10	0.02
155:	246	9.47e5	0.16	0.03
156:	247	6.34e5	0.10	0.02
157:	248	1.70e6	0.28	0.05
158:	249	3.88e6	0.64	0.11
159:	250	4.52e6	0.74	0.13
160:	251	1.97e6	0.32	0.05
161:	252	1.41e6	0.23	0.04
162:	262	9.72e6	1.60	0.27
163:	263	1.75e6	0.29	0.05
164:	264	6.83e6	1.12	0.19
165:	265	1.18e6	0.19	0.03
166:	266	1.05e6	0.17	0.03
167:	276	1.07e6	0.18	0.03
168:	290	2.57e6	0.42	0.07
169:	292	8.49e5	0.14	0.02
170:	304	9.23e5	0.15	0.03
171:	305	2.69e7	4.41	0.75
172:	306	4.50e6	0.74	0.13
173:	307	1.00e7	1.64	0.28
174:	308	1.53e6	0.25	0.04

Compound 8g

, 08-Jan-2014 + 03:19:38
Scan EI+
3.80e8

1 2809 (17.149) Cm (2779:2815-(2691:2780+2814:2846))

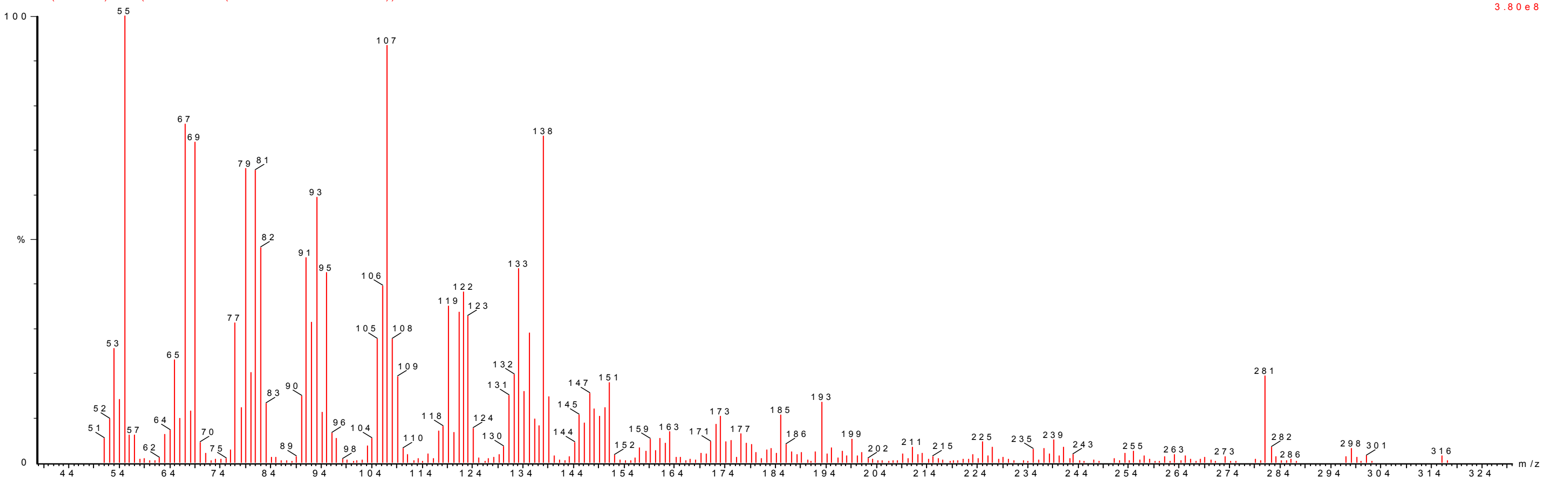

, 08-Jan-2014 + 03:19:38
Scan EI+

1 2809 (17.149) Cm (2779:2815-(2691:2780+2814:2846))

No	Mass	Inten	%BPI	%TIC
1:	51	1.99e7	5.24	0.28
2:	52	3.67e7	9.65	0.52
3:	53	9.68e7	25.44	1.38
4:	54	5.30e7	13.93	0.75
5:	55	3.80e8	100.00	5.42
6:	56	2.28e7	5.99	0.32
7:	57	2.30e7	6.05	0.33
8:	58	2.33e6	0.61	0.03
9:	59	2.88e6	0.76	0.04
10:	60	9.02e5	0.24	0.01
11:	61	1.13e6	0.30	0.02
12:	62	3.59e6	0.94	0.05
13:	63	2.35e7	6.19	0.34
14:	64	2.71e7	7.11	0.39
15:	65	8.74e7	22.96	1.24
16:	66	3.71e7	9.76	0.53
17:	67	2.88e8	75.72	4.10
18:	68	4.33e7	11.37	0.62
19:	69	2.73e8	71.73	3.89
20:	70	1.66e7	4.37	0.24
21:	71	7.37e6	1.94	0.10
22:	72	1.07e6	0.28	0.02
23:	73	2.04e6	0.54	0.03
24:	74	2.42e6	0.64	0.03
25:	75	3.09e6	0.81	0.04
26:	76	1.02e7	2.69	0.15
27:	77	1.19e8	31.19	1.69
28:	78	4.64e7	12.19	0.66
29:	79	2.50e8	65.77	3.56
30:	80	7.64e7	20.08	1.09
31:	81	2.49e8	65.34	3.54
32:	82	1.83e8	48.05	2.60
33:	83	4.96e7	13.03	0.71
34:	84	4.01e6	1.05	0.06
35:	85	3.74e6	0.98	0.05
36:	86	9.08e5	0.24	0.01
37:	87	9.91e5	0.26	0.01
38:	88	5.90e5	0.16	0.01
39:	89	4.61e6	1.21	0.07
40:	90	5.63e7	14.78	0.80
41:	91	1.74e8	45.75	2.48
42:	92	1.19e8	31.37	1.70
43:	93	2.26e8	59.30	3.21
44:	94	4.26e7	11.21	0.61
45:	95	1.62e8	42.46	2.30
46:	96	2.45e7	6.43	0.35
47:	97	1.98e7	5.21	0.28
48:	98	2.16e6	0.57	0.03
49:	99	1.49e6	0.39	0.02
50:	100	8.59e5	0.23	0.01
51:	101	9.16e5	0.24	0.01
52:	102	1.91e6	0.50	0.03
53:	103	1.35e7	3.55	0.19
54:	104	1.99e7	5.22	0.28
55:	105	1.05e8	27.56	1.49
56:	106	1.50e8	39.42	2.14
57:	107	3.55e8	93.38	5.06
58:	108	1.05e8	27.54	1.49
59:	109	7.28e7	19.13	1.04
60:	110	1.15e7	3.02	0.16
61:	111	6.19e6	1.63	0.09
62:	112	1.24e6	0.33	0.02
63:	113	2.69e6	0.71	0.04
64:	114	7.35e5	0.19	0.01
65:	115	6.80e6	1.79	0.10
66:	116	3.08e6	0.81	0.04
67:	117	2.66e7	7.00	0.38
68:	118	3.03e7	7.96	0.43
69:	119	1.33e8	34.98	1.89
70:	120	2.54e7	6.68	0.36
71:	121	1.28e8	33.54	1.82
72:	122	1.45e8	38.04	2.06
73:	123	1.24e8	32.63	1.77
74:	124	2.87e7	7.55	0.41
75:	125	3.54e6	0.93	0.05
76:	126	6.87e5	0.18	0.01
77:	127	2.70e6	0.71	0.04
78:	128	3.92e6	1.03	0.06
79:	129	6.38e6	1.68	0.09
80:	130	1.29e7	3.40	0.18
81:	131	5.67e7	14.91	0.81
82:	132	7.46e7	19.61	1.06
83:	133	1.65e8	43.33	2.35
84:	134	5.99e7	15.74	0.85
85:	135	1.10e8	28.87	1.56
86:	136	3.68e7	9.68	0.52
87:	137	3.11e7	8.18	0.44
88:	138	2.78e8	73.08	3.96
89:	139	5.55e7	14.59	0.79
90:	140	5.02e6	1.32	0.07
91:	141	1.65e6	0.43	0.02
92:	142	1.29e6	0.34	0.02
93:	143	4.35e6	1.14	0.06
94:	144	1.64e7	4.32	0.23
95:	145	4.00e7	10.51	0.57
96:	146	3.35e7	8.80	0.48
97:	147	5.86e7	15.40	0.83
98:	148	4.52e7	11.89	0.64
99:	149	3.92e7	10.31	0.56
100:	150	4.64e7	12.18	0.66
101:	151	6.77e7	17.80	0.96
102:	152	5.56e6	1.46	0.08
103:	153	1.65e6	0.43	0.02
104:	154	1.08e6	0.28	0.02
105:	155	1.16e6	0.30	0.02
106:	156	3.48e6	0.92	0.05
107:	157	1.23e7	3.23	0.17
108:	158	9.41e6	2.47	0.13
109:	159	1.94e7	5.10	0.28
110:	160	9.62e6	2.53	0.14
111:	161	2.00e7	5.25	0.28
112:	162	1.62e7	4.25	0.23
113:	163	2.56e7	6.73	0.36
114:	164	3.90e6	1.03	0.06
115:	165	4.21e6	1.11	0.06
116:	166	1.38e6	0.36	0.02
117:	167	2.52e6	0.66	0.04
118:	168	1.90e6	0.50	0.03
119:	169	7.63e6	2.00	0.11
120:	170	6.61e6	1.74	0.09
121:	171	1.71e7	4.49	0.24
122:	172	3.21e7	8.45	0.46
123:	173	3.88e7	10.20	0.55
124:	174	1.70e7	4.47	0.24
125:	175	1.84e7	4.83	0.26
126:	176	4.00e6	1.05	0.06
127:	177	2.38e7	6.25	0.34
128:	178	1.62e7	4.26	0.23
129:	179	1.49e7	3.91	0.21
130:	180	8.26e6	2.17	0.12
131:	181	2.75e6	0.72	0.04
132:	182	1.02e7	2.67	0.14
133:	183	1.13e7	2.97	0.16
134:	184	7.43e6	1.95	0.11
135:	185	4.03e7	10.60	0.57
136:	186	1.51e7	3.97	0.22
137:	187	8.72e6	2.29	0.12
138:	188	6.41e6	1.69	0.09
139:	189	7.88e6	2.07	0.11
140:	190	1.81e6	0.48	0.03
141:	191	7.89e5	0.21	0.01
142:	192	8.57e6	2.25	0.12
143:	193	5.10e7	13.41	0.73
144:	194	6.74e6	1.77	0.10
145:	195	1.18e7	3.11	0.17
146:	196	3.52e6	0.93	0.05
147:	197	9.23e6	2.42	0.13
148:	198	5.44e6	1.43	0.08
149:	199	1.95e7	5.12	0.28
150:	200	4.93e6	1.30	0.07
151:	201	8.21e6	2.16	0.12
152:	202	2.66e6	0.70	0.04
153:	203	2.01e6	0.53	0.03
154:	204	9.93e5	0.26	0.01
155:	205	1.27e6	0.33	0.02
156:	206	6.25e5	0.16	0.01
157:	207	9.70e5	0.26	0.01
158:	208	1.11e6	0.29	0.02
159:	209	6.81e6	1.79	0.10
160:	210	2.87e6	0.75	0.04
161:	211	1.24e7	3.26	0.18
162:	212	6.58e6	1.73	0.09
163:	213	7.21e6	1.90	0.10
164:	214	2.42e6	0.64	0.03
165:	215	4.81e6	1.27	0.07
166:	216	2.89e6	0.76	0.04
167:	217	1.83e6	0.48	0.03
168:	219	6.57e5	0.17	0.01
169:	219	1.06e6	0.28	0.02
170:	220	9.32e5	0.24	0.01
171:	221	2.54e6	0.67	0.04
172:	222	2.01e6	0.53	0.03
173:	223	6.07e6	1.59	0.09
174:	224	2.84e6	0.75	0.04
175:	225	1.73e7	4.56	0.25
176:	226	5.18e6	1.36	0.07
177:	227	1.26e7	3.31	0.18
178:	228	2.51e6	0.66	0.04
179:	229	3.74e6	0.98	0.05
180:	230	2.47e6	0.65	0.04
181:	231	9.97e5	0.26	0.01
182:	233	1.01e6	0.27	0.01
183:	234	6.70e5	0.18	0.01
184:	235	1.09e7	2.86	0.16
185:	236	1.78e6	0.47	0.03
186:	237	1.15e7	3.02	0.16
187:	238	6.61e6	1.74	0.09
188:	239	1.90e7	4.99	0.27
189:	240	5.41e6	1.42	0.08
190:	241	1.28e7	3.37	0.18
191:	242	3.06e6	0.80	0.04
192:	243	6.13e6	1.61	0.09
193:	244	1.32e6	0.35	0.02
194:	245	8.11e5	0.21	0.01
195:	247	1.79e6	0.47	0.03
196:	248	5.62e5	0.15	0.01
197:	251	2.81e6	0.74	0.04
198:	252	1.01e6	0.27	0.01
199:	253	7.46e6	1.96	0.11
200:	254	1.07e6	0.28	0.02
201:	255	8.89e6	2.34	0.13
202:	256	1.88e6	0.49	0.03
203:	257	4.96e6	1.30	0.07
204:	258	2.24e6	0.59	0.03
205:	259	7.36e5	0.19	0.01
206:	260	4.27e5	0.11	0.01
207:	261	4.49e6	1.18	0.06
208:	262	5.60e5	0.15	0.01
209:	263	6.51e6	1.71	0.09
210:	264	1.30e6	0.34	0.02
211:	265	5.13e6	1.35	0.07
212:	266	2.30e6	0.60	0.03
213:	267	8.32e5	0.22	0.01
214:	268	2.15e6	0.57	0.03
215:	269	4.10e6	1.08	0.06
216:	270	1.66e6	0.44	0.02
217:	271	8.03e5	0.21	0.01
218:	273	4.34e6	1.14	0.06
219:	274	7.26e5	0.19	0.01
220:	275	6.23e5	0.16	0.01
221:	279	2.13e6	0.56	0.03
222:	280	1.20e6	0.32	0.02
223:	281	7.35e7	19.32	1.05
224:	282	1.29e7	3.38	0.18
225:	283	4.69e6	1.23	0.07
226:	284	9.92e5	0.26	0.01
227:	285	1.10e6	0.29	0.02
228:	286	2.17e6	0.57	0.03
229:	287	6.57e5	0.17	0.01
230:	297	4.48e6	1.18	0.06
231:	298	1.14e7	2.99	0.16
232:	299	4.11e6	1.08	0.06
233:	300	5.46e5	0.14	0.01
234:	301	4.90e6	1.29	0.07
235:	302	7.58e5	0.20	0.01
236:	316	5.38e6	1.41	0.08
237:	317	9.07e5	0.24	0.01
